# Design, Synthesis and Pharmacological Evaluation of Three Novel Dehydroabietyl Piperazine Dithiocarbamate Ruthenium (II) Polypyridyl Complexes as Potential Antitumor Agents: DNA Damage, Cell Cycle Arrest and Apoptosis Induction

**DOI:** 10.3390/molecules26051453

**Published:** 2021-03-07

**Authors:** Haoran Wang, Jianhua Wei, Hong Jiang, Ye Zhang, Caina Jiang, Xianli Ma

**Affiliations:** 1 School of Pharmacy, Guilin Medical University, Guilin 541199, China; wk811726746@163.com (H.W.); jh931209@163.com (H.J.); zhangye81@126.com (Y.Z.); 2 Department of Chemistry & Pharmaceutical Science, Guilin Normal College, Xinyi Road 15, Guilin 541001, China

**Keywords:** dithiocarbamate, ruthenium polypyridyl complexes, antitumor activity, DNA damage, cell cycle arrest, apoptosis

## Abstract

The use of cisplatin is severely limited by its toxic side-effects, which has spurred chemists to employ different strategies in the development of new metal-based anticancer agents. Here, three novel dehydroabietyl piperazine dithiocarbamate ruthenium (II) polypyridyl complexes (**6a**–**6c**) were synthesized as antitumor agents. Compounds **6a** and **6c** exhibited better in vitro antiproliferative activity against seven tumor cell lines than cisplatin, they displayed no evident resistance in the cisplatin-resistant cell line A549/DPP. Importantly, **6a** effectively inhibited tumor growth in the T-24 xenograft mouse model in comparison with cisplatin. Gel electrophoresis assay indicated that DNA was the potential targets of **6a** and **6c**, and the upregulation of p-H2AX confirmed this result. Cell cycle arrest studies demonstrated that **6a** and **6c** arrested the cell cycle at G1 phase, accompanied by the upregulation of the expression levels of the antioncogene p27 and the down-regulation of the expression levels of cyclin E. In addition, **6a** and **6c** caused the apoptosis of tumor cells along with the upregulation of the expression of Bax, caspase-9, cytochrome c, intracellular Ca^2+^ release, reactive oxygen species (ROS) generation and the downregulation of Bcl-2. These mechanistic study results suggested that **6a** and **6c** exerted their antitumor activity by inducing DNA damage, and consequently causing G1 stage arrest and the induction of apoptosis.

## 1. Introduction

DNA is one of the major targets for antitumor drugs and plays an important role in the division and growth of uncontrolled cells. A great deal of efforts has been devoted to the design and synthesis of antitumor agents targeting DNA. Many commercial anticancer drugs, including cisplatin, mitomycin, fluorouracil and 2-aminoadenine, inhibit transcription by targeting DNA, thus leading to DNA damage and consequently triggering cell cycle checkpoint and apoptosis responses [1,2,3,4].

Platinum-based anticancer drugs containing cisplatin, carboplatin and oxaliplatin have been widely and clinically used for the treatment of testicular and ovarian cancer and a range of other malignancies [5,6]. Although platinum-based drugs exhibit effective antitumor effects, their applications are limited by some severe disadvantages, such as nephrotoxicity, neuro-toxicity and intrinsic/ acquired resistance [7]. To improve these disadvantages, alternative metal-based drugs that lead to antitumor mechanisms different from platinum-based chemotherapy agents have been explored and proved be a feasible strategy [8,9]. In particular, ruthenium-based antitumor complexes have gained a great deal of interest due to their promising antitumoral and antimetastatic properties [10,11,12]. Two leading types of ruthenium complexes, imidazolium trans-[tetrachloro(S-dimethyl- sulfoxide)(1H-imida zole) ruthenate (III)] (NAMI-A) and indazolium [trans-tetrachlorobis (1H-indazole) ruthenate (III)] (KP1019) have entered phase II clinical trials to treat cancer [12,13,14]. The discovery of NAMI-A and KP1019 triggered a great deal of study on the design and synthesis of ruthenium-based antitumor complexes. The anticancer mechanism of these complexes have also been well documented, which include disrupting the binding of transcription factors to DNA, triggering DNA damage, arresting the cell cycle, causing mitochondrial dysfunction, etc. [15,16,17,18,19].

It is believed that the introduction of natural active ingredients as ligands is a feasible method to improve the toxicity and resistance of platinum-based antitumor drugs [4,20]. Pentacyclic triterpenes have been recognized as natural pharmacophores that exhibit various biological activities, including antitumor and antimetastatic activity. Dehydroabietic acid (DHAA) is a natural pentacyclic triterpene that exhibits a wide range of biological activities, such as antiviral, anxiolytic, antimicrobial and antitumor activities [21,22,23,24,25]. Many sources of evidence have verified that DHAA and its analogs display potent antiproliferative activity against some human cancer cells, including hepatocellular carcinoma, gastric cancer and breast cancer cells [26]. In addition, many potent antitumor DHAA derivatives containing α-aminophosphonate, dipeptides, and thiourea have also been designed and prepared in our previous work. These compounds usually exert their antitumor activities by the inducing of cancer cell cycle arrest and apoptosis [27,28,29,30]. Our interesting in the potential antitumor activity of DHAA derivatives has encouraged us to choose DHHA as an active structure for modification and screening of antitumor agents for many years. In addition, piperazine is known as a classic scaffold usually contained in a series of clinical antitumor drugs such as entrectinib, olmutinib and palbociclib which is believed to increase the antitumor properties. Moreover, dithiocarbamato was created as a special ligand that exhibits strong coordination ability with various transition metals to improve the antitumor effects, while many dithiocarbamate complexes such as [Cu(DMDT)_2_], mer-[RuCl_3_(DMSO)(DMDTM)] and [AuCl_2_(MSDT)] display good antitumor efficiency [31]. Inspired by the antitumor medicinal virtue of DHHA, piperazine, dithiocarbamate and ruthenium complexes, it is thus expected that the combination of DHHA, piperazine, dithiocarbamate and ruthenium may offer some functional complexes with good antitumor efficiencies and improved toxicities. Therefore, in the present work, as a continuation of our previous work [28,29,30], a novel DHHA-piperazine-dithiocarbamate ligand was designed and synthesized to coordinate with ruthenium polypyridyl complexes to offer three novel DHHA-piperazine-dithiocarbamate ruthenium polypyridyl complexes. We expected that the combination of DHHA, piperazine, dithiocarbamate and ruthenium polypyridyl would trigger positive synergistic effects, thereby improving the antitumor efficiency, toxicity and resistance. With this expectation in mind, the target complexes, their antiproliferative activities and their antitumor mechanisms related to DNA-damage were investigated.

## 2. Results and Discussion

### 2.1. Chemistry

**Scheme 1:**
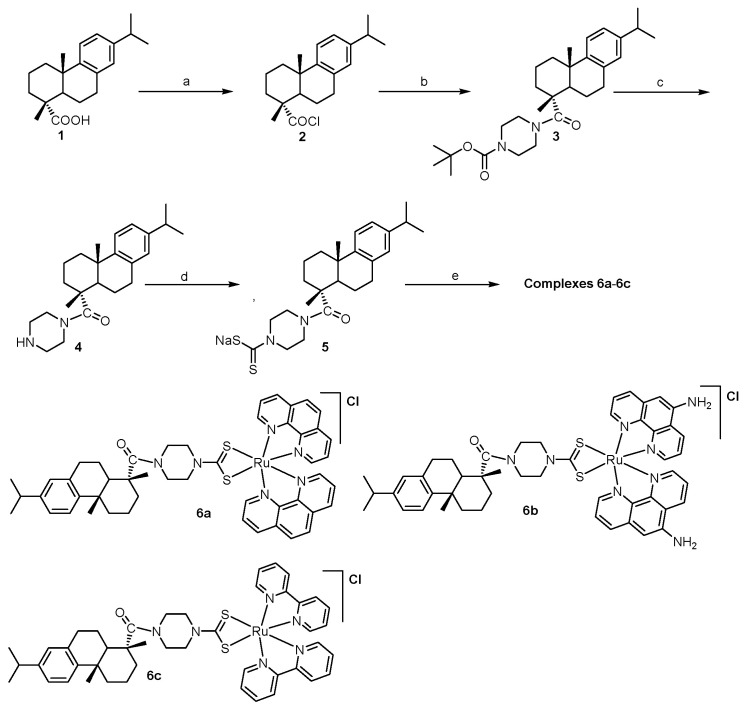
Synthetic routes of complexes **6a**–**6c**. Reagents and conditions: (a) C_2_Cl_2_O_2,_ DMF; (b) Boc-piperazine; (c) trifluoroacetic acid (TFA); (d) CS_2_, NaOH; (e) phenanthroline ruthenium dichloride / tris(1,10-phenanthroline) ruthenium (II) dichloride(A) / tris (5-diamino-1,10-phenanthroline) ruthenium dichloride(B) / tris(2,2′-bipyridine)ruthenium (II) dichloride (C).

The synthetic route of the target DHHA-piperazine-dithiocarbamate ruthenium complexes **6a**–**6c** is shown in Scheme 1. As shown in Scheme 1, DHHA (1) was firstly treated with oxalyl chloride to form dehydroabietic acid chloride (2) at 0 °C in the presence of dry *N*,*N*-dimethylformamide (DMF) [27,28,29,30], which was then treated with *t*-Butyloxy carbonyl (Boc)-piperazine to form a good yield of DHHA-Boc-piperazine (3) at room temperature in the presence of dichloromethane. The treatment of compounds (3) and trifluoroacetate (TFA) at room temperature formed a good yield of DHHA-piperazine (4) in the presence of dichloromethane. Finally, the three DHHA-piperazine-dithiocarbamate ruthenium complexes **6a**–**6c** were obtained in moderate yields by the coordination reaction of compound (4) with tris(1,10-phenanthroline) ruthenium (II) dichloride(A), tris (5-diamino-1,10-phenanthro- line) ruthenium dichloride(B) and tris(2,2′-bipyridine) ruthenium (II) dichloride (C), respectively. The chemical structures of compounds **3**–**5** and complexes **6a**–**6c** were then identified by ^1^H NMR, ^13^C NMR, high-resolution mass spectrometry (HR–MS) or/and elemental analysis (EA) (Seen in Part 1.1 of Supplementary Data).

For compounds **3**–**5**, in the ^1^H NMR spectra the chemical shift (δ) in the range of 6.88–7.20 were ascribed to the aromatic hydrogens (H-Ar) fused in the DHHA moiety, and the two broad peaks around δ 3.39 and 3.65 were attributed to the hydrogens of methylene in the piperazine ring, respectively, while δ in the range of 1.20–1.50 were mainly ascribed to the hydrogens of the methyl group in the DHHA and Boc groups. In the ^13^C NMR spectra, δ around 177 was mainly attributed to the carbons in carbonyl groups in DHHA moiety, and δ around 46 and 53 were attributed to the carbons in the piperazine moiety, while δ at 196 was attributed to the carbon in the thiocarbonyl group. In addition, the HR–MS results of **3**–**5** were also consistent with their chemical structures in Scheme 1.

For complexes **6a**–**6c**, in the ^1^H NMR spectra the peaks in the range of 7.20–9.60 were contributed by the aromatic hydrogens in the polypyridyl ruthenium moiety, while other hydrogen peaks were consistent with that of ligand **5**, indicating the coordination of ligand **5** with polypyridyl ruthenium. In the ^13^C NMR spectra, δ at 214.28 and 177.70 were ascribed to the carbons in the thiocarbonyl and carbonyl groups, respectively, while δ in the range of 123–158 was attributed to the aromatic carbon in the polypyridyl moiety group and the DHHA moiety, confirming again the coordination effect of ligand **5** with polypyridyl ruthenium. In the HR-MS analysis, complexes **6a** [C_49_H_51_N_6_ORuS_2_]^+^, **6b** [C_49_H_53_N_8_ORuS_2_]^+^ and **6c** [C_45_H_51_N_6_O RuS_2_]^+^ were accurately confirmed with the peaks at 905.2605, 935.20198 and 857.26068, respectively, confirming the structure of complexes **6a**–**6c**. The EA data of complexes **6a**–**6c** were also well consistent with that of NMR and HR-MS, fully proving the structures of complexes **6a**–**6c**.

On the basis of the NMR (^1^H and ^13^C), HR-MS and EA results, the chemical structures of compounds **3–5** and complexes **6a**–**6c** were identified. The stability of complexes **6a**–**6c** in methanol-water was then examined by high-performance liquid chromatography (HPLC) at different time points. The results (Figure S3) indicated that complexes **6a**–**6c** mainly exhibited one main peak in HPLC at 0 h, 12 h, and 24 h, respectively, implying that complexes **6a**–**6c** had good stability in the CH_3_OH/H_2_O (30:70) solution.

### 2.2. Antiproliferative Activity

#### 2.2.1. In Vitro Antiproliferative Activity

To investigate the antiproliferative activity and resistance of complexes **6a**–**6c**, 3-(4,5-dimethyl-2-thiazolyl)-2,5-diphenyl-2-*H*-tetrazolium bromide (MTT) assays were carried out against human gastric cancer cell line MGC-803, human bladder cancer cell line T-24, human liver cancer cells HepG2, human nasopharyngeal cancer cells CNE2, human breast cancer cell line MDA-MB-231, human breast cancer cells MCF-7, human hepatoma cell line A549 and its cisplatin-resistant cell line A549/CDDP, using the clinical anticancer drug cisplatin as positive control for comparison.

As shown in Table 1, complexes **6a** and **6c** exhibited significantly better antiproliferative activity than **6b**, ligand **5** and three corresponding ruthenium pyridinium chloride salts **A**–**C**, respectively. By comparing the in vitro antiproliferative activity of the ligand DHHA-piperazine-dithiocarbamate with its complexes **6a** and **6c**, it may be concluded that 2,2′-bipyridine and 1,10-phenanthroline moieties exhibited similar positive effect on their antitumor activity. With regard to **6b**, this positive effect failed, and **6b** displayed low anticancer activity similar to that of ligand **5** and its corresponding ruthenium pyridinium chloride salt **B.** The lack of inhibition of cancer cells may attribute to the low cellular uptake efficiencies as shown in the test of the subsequent concentrations in T-24 cells.

It was noteworthy that **6a** and **6c** showed higher antiproliferative activity than cisplatin on all the selected cancer cell lines, with IC_50_ values in the range of 1.0 ± 0.2–4.2 ± 0.7 μM, respectively. These results validated that inducing DHHA-piperazine-dithiocarbamate into ruthenium pyridinium chloride salts to form six coordinated cationic complexes is an effective method to prepare high-activity antitumor agents.

Table 2 shows that cisplatin exhibited evident and serious resistance in A549/CDDP cells, with IC_50_ of 30.5 ± 0.2μM, in comparison with that of 6.4 ± 1.0 μM in A549 cells. It is worth noting that **6a** and **6c** displayed no evident resistance in A549/CDDP cells. The IC_50_ for complexes **6a** and **6c** in A549/CDDP cells were 1.31 ± 1.39 μM and 1.75 ± 1.43 μM, respectively, which were similar to that in the A549 cells. The results confirmed that the combination of DHHA, piperazine, dithiocarbamate and ruthenium polypyridyl may indeed improve resistance.

#### 2.2.2. In Vivo Antiproliferative Activity

The antitumoral activity of the complex was then assessed for 27 days by treating mice with **6a** at two doses (6/12 mg kg^−1^) by tail vein injection once every three days. Specific pathogen-free BALB/c nude mice (both male and female) were divided randomly into four groups (*n* = 4), i.e., the vehicle control group, the low-dose and high-dose administration of **6a** groups and the positive control groups.

**Figure 1:**
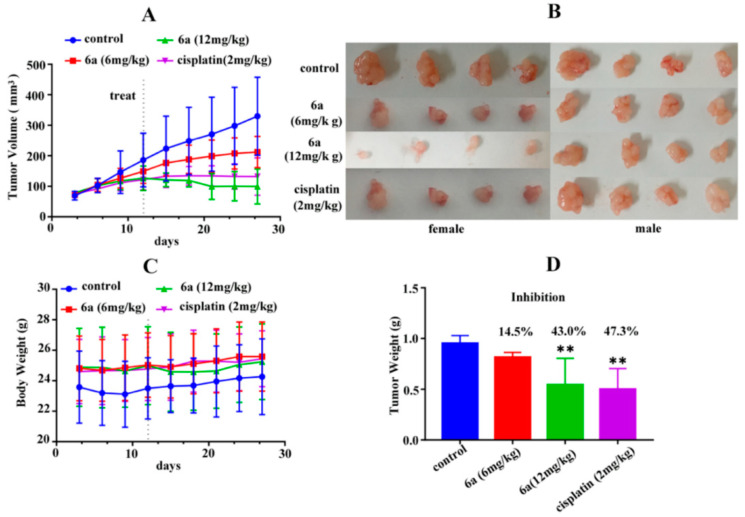
In vivo antitumor activity of **6a** in T-24 xenograft. (**A**) **6a** (at 6 and 12 mg/kg doses), cisplatin (at 2 mg/kg dose), or vehicle (5% DMSO in saline, *v*/*v*) was administered by tail vein injection to inhibit the tumor growth. Tumor growth was monitored by the mean tumor volume (mm^3^) ± SD (*n* = 4) and calculated as the relative tumor increment rate (T/C, %). (**B**) Photographs of the harvested tumors from the mice (**C**) Body weight change in the mice treated with **6a**. (**D**) Tumor weight of the mice. The tumors were collected in the mice at day 27(** *p* < 0.01).

On day 27, the average tumor volume was 318 mm^3^ for the control group and 172 mm^3^ and 152 mm^3^ for low-dose and high-dose administration of **6a**, which were 46% and 52% lower than that of the control group **6a**, with the inhibition ratio of 43.0% (*p* < 0.001) at high-doses, exhibited equivalent suppression of tumor growth to cisplatin (47.3%, *p* < 0.001) (Figure 1D). This result indicated that complex 6a may be a good candidate for antitumor agents. 

#### 2.2.3. Concentration in T-24 Cells of **6a** and **6c**

The intracellular ruthenium concentrations of **6a** and **6c** in whole cells were then investigated by inductively coupled plasma mass spectrometry (ICP–MS) with T-24 cells.

As shown in Figure S1, the order for the dose of these three ruthenium complexes T-24 cells was as follows: **6c** > **6a** > **6b**, while **6b** even did not penetrate into the cells. Combined with the MTT results, it was concluded that the sharply elevated cellular levels of ruthenium complexes might be one of the main reasons for the remarkable antiproliferative activity of **6a** and **6c**.

### 2.3. Antitumor Mechanism

#### 2.3.1. DNA Intercalation and Damage

**Figure 2:**
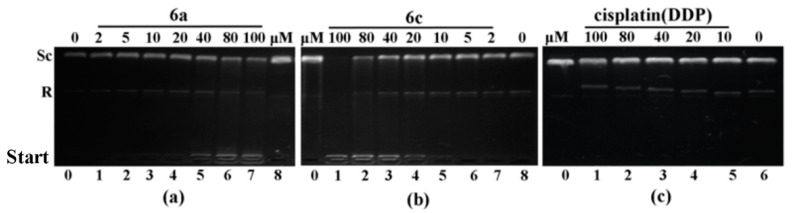
Gel electrophoresis mobility shift assay of pBR322 DNA treated with 6a, 6c and cisplatin. (**a**) Lane 0: DNA alone; lanes 1–7: DNA + 6a; lane 8: DNA+DMSO; (**b**) lane 0: DNA + DMSO; lanes 1–7: DNA + 6c; lane 8: DNA alone; (**c**) lane 0: DNA + DMSO; lanes 1–5: DNA + cisplatin; lane 6: DNA alone; R: linear from DNA, Sc: supercoiled DNA.

A planar chromophore moiety is the common feature of DNA-intercalating anticancer drugs [32] and DNA is the potential target of ruthenium (II) polypyridyl complexes [33]. It is thus assumed that DNA may be potential target for **6a** and **6c**. To investigate the DNA-intercalation ability of **6a** and **6c**, a gel electrophoresis assay was performed with cisplatin as the positive controls. Gel electrophoresis assay results (Figure 2) demonstrated that **6a** and **6c** tightly bound to the supercoiled circular plasmid pBR322 and hindered its migration in the gel at concentrations from 10 μM to 100 μM, while cisplatin bound to its circular form and hindered its migration at the same concentrations, indicating that **6a** and **6c** exhibited important intercalation effects on pBR322.

**Figure 3:**
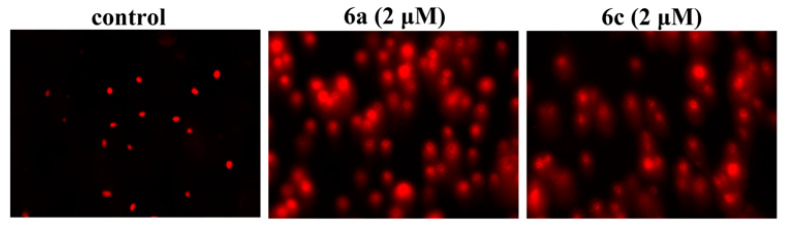
Images of comet assay of T-24 cells after treatment with **6a** and **6c** for 18 h.

**Figure 4:**
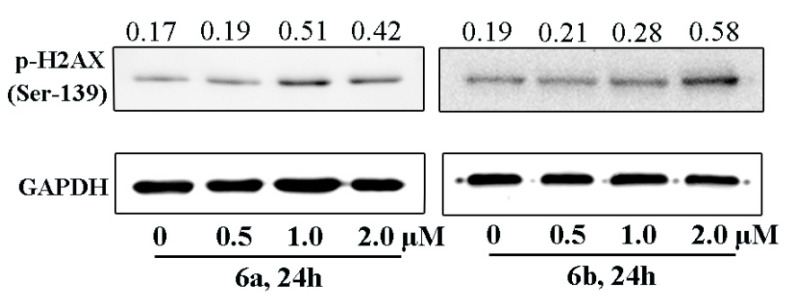
The expression of DNA damage-associated proteins phosphor-H2AX in T-24 cells after incubation with the indicated concentrations of **6a** and **6c**.

To further investigate whether **6a** and **6c** could lead to DNA damage, the comet assay was performed and the expression level of p-H2AX was examined by Western blots. Figure 3 showed that the treatment with **6a** and **6c** led to long DNA tails, implying that **6a** and **6c** could induce DNA damage [34]. In addition, the treatment of **6a** and **6c** resulted in the upregulation of p-H_2_AX expression level (Figure 4), a well-known marker for DNA double-strand breaks, further confirming the presence of DNA damage [32].

#### 2.3.2. Cell Cycle Arrest Analysis

It is known that the damage of DNA could initiate cell cycle arrest [2]. To investigate whether **6a** and **6c** could induce cell cycle progression, cell cycle arrest assay was carried out with T-24 cells. The cell cycle arrest assay results (Figure S2) indicated that **6a** and **6c** mainly arrested the cell cycle at G1 the phase, leading to an evident increase in the G1 phase population (for **6a**: 64.62% at 0.5 μM, 61.64% at 1 μM and 71.98% at 2 μM; for **6c**: 56.24% at 0.5 μM, 64.11% at 1 μM and 62.77% at 2 μM) in comparison with the control group (55.31%).

**Figure 5:**
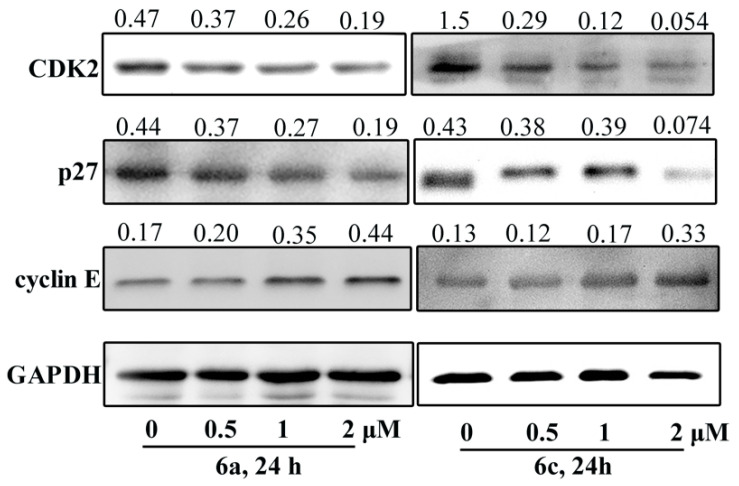
Effects of **6a** and **6c** treatment in T-24 cells on cell cycle regulatory proteins at 0.5, 1 and 2 μM for 24 h, respectively.

The regulatory proteins CDK and cyclin E, as well as antioncogene p27, play an important regulated role in G1 phase checkpoints [35]. The expression of CDK2, cyclin E and p27 in T-24 cells was tested by Western blots assays, using glyceraldehyde-3-phosphate dehydrogenase (GAPDH) as a control. The Western blot results (Figure 5) demonstrated that treatment with **6a** and **6c** increased cyclin E expression and decreased p27 and CDK2 expression, indicating that **6a** and **6c** induced G1 phase cell cycle arrest by inhibiting the G1 phase-promoting CDK2–cyclin E complex in T-24 cells [36].

#### 2.3.3. Apoptosis: Mode of Cell Death Induced by Compounds **6a** and **6c**

**Figure 6:**
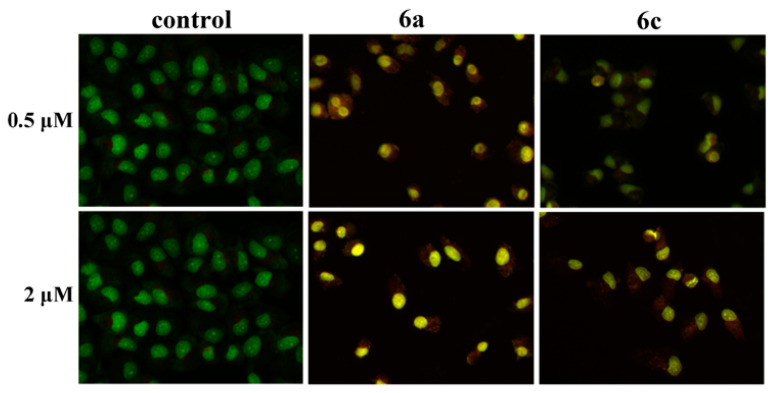
T-24 cells were stained with AO and observed with an EVOS M5000 Imaging System. T-24 cells in the absence (control group) and presence of different concentrations of **6a** and **6c**, incubated at 37 °C and 5% CO_2_/95% air in a humidified incubator for 12 h, are shown.

**Figure 7:**
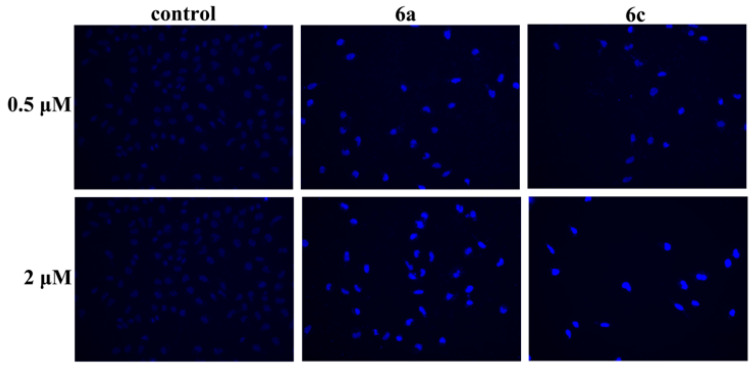
T-24 cells were stained with Hochest 33258 and observed using an EVOS M5000 Imaging System. T-24 cells in the absence (control group) and presence of different concentrations of **6a** and **6c**, incubated at 37 °C and 5% CO_2_/95% air in a humidified incubator for 24 h, are shown.

Since DNA damage can initiate an apoptosis response [1,2,3,4], the effect of **6a** and **6c** on apoptosis progression was examined. Acridine orange (AO) staining assay results (Figure 6) showed that the morphology of **6a**- and **6c**-treated T-24 cells exhibited clear changes and that cell nuclei were stained as yellow/orange, indicating chromatin condensation or breakage caused by apoptosis, and that **6a** and **6c** could induce apoptosis in T-24 cells. In addition, as Figure 7 shows, Hoechst 33258 staining of T-24 cells treated with **6a** and **6c** exhibited strong blue fluorescence and typical apoptotic morphologies at the concentrations 0.5 and 2 µM, respectively, while T-24 cells not treated with **6a** and **6c** displayed normal blue fluorescence, also confirming that **6a** and **6c** could indeed induce apoptosis in T-24 cells.

**Figure 8:**
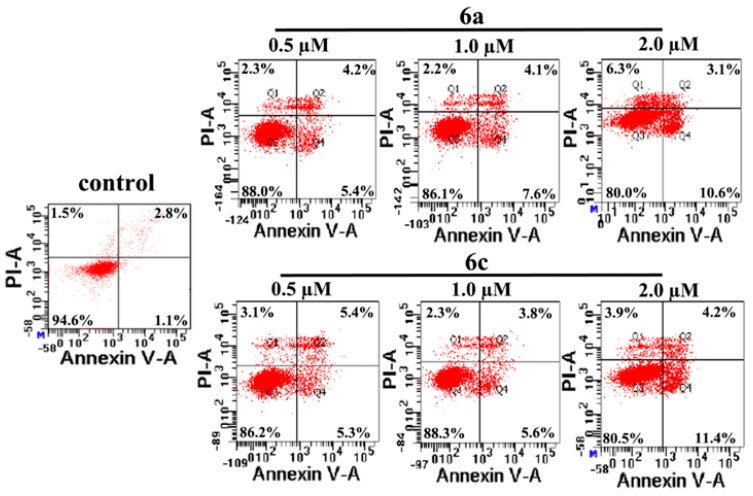
Induction of apoptosis by **6a** and **6c** in the T-24 cell line. The cells were treated with the complexes for 24 h, and their apoptosis was determined using Annexin V/propidium iodide (PI) staining and a flow cytometer. Annexin V (+) and PI (−) cells are labelled as early apoptotic cells, whereas Annexin V (+) and PI (+) cells are labelled as late apoptotic cells.

The apoptotic cell rates were then determined with the T-24 cells treated with **6a** and **6c** at the concentration of 0, 0.5, 1 and 2 for 24 h, respectively. The results (Figure 8) demonstrated that treatment of the T-24 cells with complexes **6a** and **6c** at different concentrations led to obvious increases of the apoptotic cell population (9.6%, 11.7%, 13.7% and 10.7%, 9.4%, 15.6%, respectively), in comparison with the control (3.9%), implying that **6a** and **6c** could induce the apoptosis of T-24 cells.

**Figure 9:**
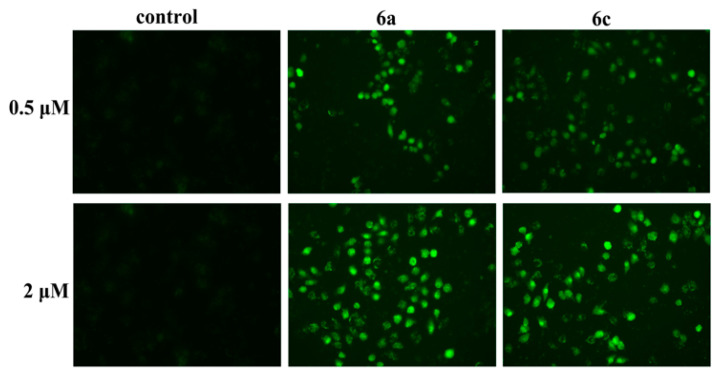
Effect on intracellular free Ca^2+^ in T-24 cells after treatment with **6a**/**6c** at 0.5 and 2 μM for 24h. Fluo-4 AM was used as the fluorescent probe to determine the concentration of intracellular free Ca^2+^.

**Figure 10:**
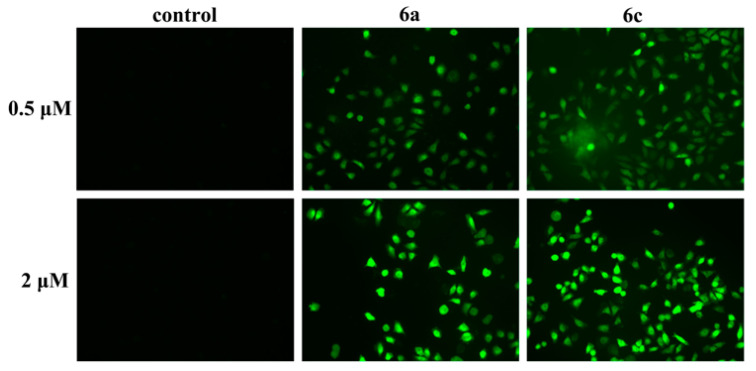
**6a** and **6c** showed evident induction of intracellular ROS in T-24 cells. Cells were treated with the indicated concentration of complexes for 3h before staining with 10 μM DCF-DA for 30 min.

**Figure 11:**
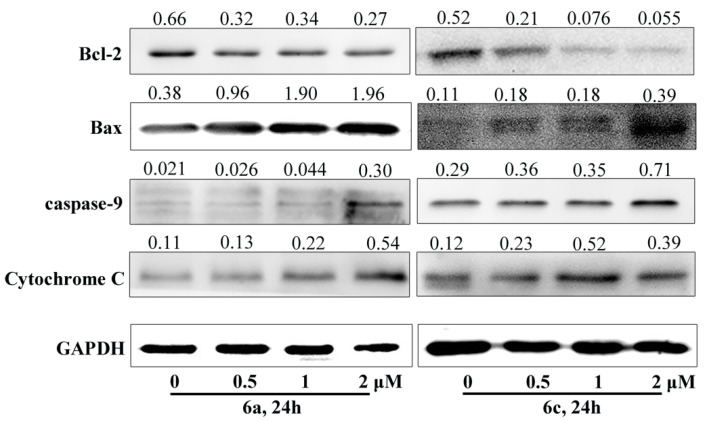
Western blot analysis of caspase-9, Bcl-2, Bax and cytochrome C after treatment of T-24 cells with **6a** and **6c** at concentrations of 0.5, 1 and 2 μM, respectively, for 24 h.

It is believed that calcium and cytochrome c significantly participate during cell apoptosis and Bcl-2/Bax family proteins, ROS and caspase-9 play important roles in regulating apoptosis. To further investigate the mechanisms underlying **6a**-and **6c**-induced apoptosis, the release of intracellular ROS and calcium ion and the expression levels of Bax, Bcl-2, cytochrome c and caspase-9 in T-24 cells treated with **6a** and **6c** were tested by fluorescence staining and Western blot assays, respectively. The fluorescence staining assay results (Figure 9 and Figure 10) showed that the morphology of **6a**- and **6c**-treated T-24 cells exhibited strong green fluorescence, while the control group displayed the normal/dark green fluorescence, indicating complexes **6a** and **6c** could significantly increase the intracellular level of calcium and ROS release. The Western blot assay results (Figure 11) demonstrated that **6a** and **6c** could upregulate the expression levels of Bax, cytochrome c and caspase-9 and downregulate Bcl-2 levels, indicating that **6a** and **6c** may exert proapoptotic effects through a mitochondria-mediated pathway and a caspase cascade.

## 3. Materials and Methods

### 3.1. Materials and Instruments

All chemicals and solvents were of reagent grade and purchased from Aladdin (Shanghai, China) and used without further purification. The materials used for biological experiments with pBR322 DNA and cell lines were purchased from Aladdin. Cell cycle and apoptosis assays were performed by BD FACSAria III flow cytometry (Becton Dickinson, Franklin Lakes, NJ, USA) and the results were analyzed by ImageJ software. The NMR spectra were measured on a BRUKER AVANCE AV 400/600 instrument (Billerica, MA, USA), while the mass spectra were examined on a BRUKER ESQUIRE HCT spectrometer.

#### 3.1.1. Chemistry: General Synthesis Procedure for Compounds **4**–**7**

Dehydroabietic acid (1) and dehydroabietic acid chloride (2) were prepared according to our previous work [18,19], using rosin as the starting material. The N-Boc-piperazine (1 mmol) in dichloromethane (15 mL) was added dropwise to the dichloromethane (30 mL) of compound 2 (1 mmol), and the mixture solution was then stirred for 6 h after dripping. By vacuum distillation, DHHA-Boc-piperazine (**3**) was obtained with a yield of 82.3%. The mixture of compounds (**3**) (1 mmol), trifluoroacetate (TFA) (10 mL) and dichloromethane (50 mL) was stirred at room temperature for 6 h. By vacuum distillation of this solution, DHHA-piperazine (**4**) was obtained with a yield of 76.5%. The mixture of DHHA-piperazine 4 (1 mmol), saturated sodium hydroxide solution (4 mL), methanol (50 mL) and carbon disulfide (2 mL) was reacted at room temperature for 6 h. After vacuum distillation, the residuals were purified by silica column chromatography using dichloromethane–methanol (*v*:*v* = 100:1) solution as the eluent to form compound (**5**) with a yield of 63.2%. 

The mixture of 5 (1 mmol), dipyridine ruthenium intermediate (1 mmol), tetramethyl ammonium hydroxide aqueous solution (1 mL), and dichloromethane-methanol solution (20 mL, volume ratio 2:1) was stirred and reacted at 65 °C for 24 h, according to the reference [37]. After the reaction, complexes **6a**–**6c** were obtained through vacuum evaporation and then purified by neutral alumina column chromatography using dichloromethane–methanol (*v*:*v* = 100:2) solution as the eluent.

#### 3.1.2. Biological Assays

The biological assays, including MTT assays, ICP–MS, gel electrophoresis, intracellular ROS and Ca^2+^, flow cytometry and Western blotting were carried out according to our previous work [38,39].

## 4. Conclusions

In this study, three novel dehydroabietyl piperazine dithiocarbamate polypyridyl-ruthenium (II) complexes were synthesized and their antitumor activity were evaluated against seven cancer cell lines, i.e., MGC-803, T-24, HepG2, CNE-2, MDA-MB-231, MCF-7 and A549. We identified that, in comparison with cisplatin, **6a** and **6c** exhibited better in vitro antitumor activity against these selected cancer cell lines, while they displayed no evident resistance in the cisplatin-resistant cell line A549/DPP. Importantly, **6a** effectively inhibited tumour growth in the T-24 xenograft mouse model. Our gel electrophoresis assay indicated that DNA is a potential target of **6a** and **6c**, and our p-H2AX assay confirmed that **6a** and **6c** could induce DNA damage. Cell cycle arrest studies indicated that complexes **6a** and **6c** trigger cell cycle arrest at the G1 phase through inhibition of CDK2–cyclin E complex activity, while apoptosis assays indicated that **6a** and **6c** initiated the apoptosis of T-24 cells accompanied by the upregulation of the expression of Bax and caspase-9 and downregulation of Bcl-2. In short, our study deduced that 6a and 6c may mainly exert their antitumor effects by inducing DNA damage, and consequently causing to G1 phase arrest and inducing apoptosis.

## Figures and Tables

**Table 1:** In vitro antitumor activity of compounds **6a**–**6c**.

	MGC-803	T-24	HepG2	CNE-2	MDA-MB-231	MCF-7
5	>50	>50	>50	>50	>50	>50
A	>50	>50	>50	>50	>50	>50
B	>50	>50	>50	>50	>50	>50
C	>50	>50	>50	>50	>50	>50
6a	1.2 ± 0.9	1.2 ± 0.7	2.0 ± 0.3	3.2 ± 0.1	3.4 ± 0.7	2.1 ± 0.6
6b	>50	>50	>50	>50	>50	>50
6c	1.6 ± 0.5	1.0 ± 0.2	2.2 ± 0.4	4.2 ± 0.7	1.3 ± 0.5	3.6 ± 0.3
Cisplatin	10.1 ± 0.2	9.5 ± 0.6	7.1 ± 0.7	>50	14.7 ± 1.9	21.5 ± 0.6

IC_50_ values are presents as mean ±SD (standard error of the mean) from three repeating experiments. Compounds were incubated with tested cancer cell lines for 48 h.

**Table 2:** IC_50_ of the ligand and its complexes **6a** and **6c** in A549 and A549-DDP cancer cell lines.

	A549	A549-DDP
5	>50	>50
A	>50	>50
B	>50	>50
C	>50	>50
6a	5.2 ± 0.6	1.3 ± 0.3
6b	>50	>50
6c	5.5 ± 0.9	1.8 ± 0.4
Cisplatin	6.4 ± 1.0	30.5 ± 0.2

## Data Availability

All data is contained within the article or supplementary material.

## Supplementary Materials

The following are available online. Figure S1: The intracellular ruthenium concentrations in T-24 cells; Figure S2: Cell cycle distribution of T-24 cells exposed to **6a** and **6c** (0.5, 1, 2 μM) for 24 h. Effects on cell cycle progression of these compounds were examined according to the procedures described in the experimental section; Figure S3: HPLC spectra for **6a**, **6b** and **6c** in aqueous solution (1 mg/mL) in the time courses of 0 h, 12 h and 24h, respectively. Column: reversed-phase C18 column (Agilent 5 TC-C18 250 × 4.6 mm.). Column temperature: 35 °C. Mobile phase: CH_3_OH/H_2_O (30:70). Flow rate: 1.0 mL/min. Injection volume: 20 μM; the ESI-MS spectrum, and ^1^H-NMR, ^13^C-NMR spectra of compounds 3–5 and complexes **6a**–**6c**.

## Sample Availability

Samples of the compounds are available from the authors.
