# Supplementary material for: Design, Synthesis and Pharmacological Evaluation of Three Novel Dehydroabietyl Piperazine Dithiocarbamate Ruthenium (II) Polypyridyl Complexes as Potential Antitumor Agents: DNA Damage, Cell Cycle Arrest and Apoptosis Induction
Source: Molecules. 2021 Mar 7;26(5):1453. doi: 10.3390/molecules26051453 (PMC7962184; doi:10.3390/molecules26051453)
Supplement: Supplementary file 1 [file molecules-26-01453-s001.pdf]

Design, synthesis and pharmacological evaluation of three novel dehydroabietyl piperazine dithiocarbamate ruthenium (II) polypyridyl complexes as potential antitumor agents: DNA damage, cell cycle arrest and apoptosis inducing

Hao-Ran Wang[1,†], Jian-Hua Wei[1,†,*], Hong Jiang[1], Ye Zhang[1,2], Cai-Na Jiang[1,*], Xian-Li Ma[1,2,*]

[1] *School of Pharmacy, Guilin Medical University, Guilin 541004, China.*

[2] *Department of Chemistry & Pharmaceutical Science, Guilin Normal College, Xinyi Road 15, Guangxi 541001, China*

[†] These authors contributed equally to this work.

E-mail addresses: 120096545@qq. com (Xian-Li Ma), 429477986@qq.com (Cai-Na Jiang)

1. Metal accumulation in T-24 cell

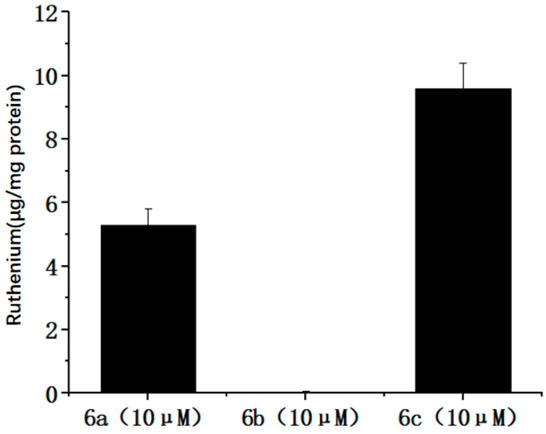

Figure S1: ICP-MS of T-24 cell uptake of complexes **6a**-**6c**. T-24 cells incubated with complexes **6a**-**6c** for 2h at 37°C in a humidified atmosphere of 5% CO_2/95% air. All cells were collected, and then resuspended in 0.5 mL of PBS solution. Ruthenium concentrations were determined by ICP-MS (Inductively-Coupled Plasma Mass Spectrometry). Samples then treated with concentrated HNO_3 overnight prior to analysis. Cellular concentrations of ruthenium were reported per μg of protein.

2. Cell cycle arrest analysis

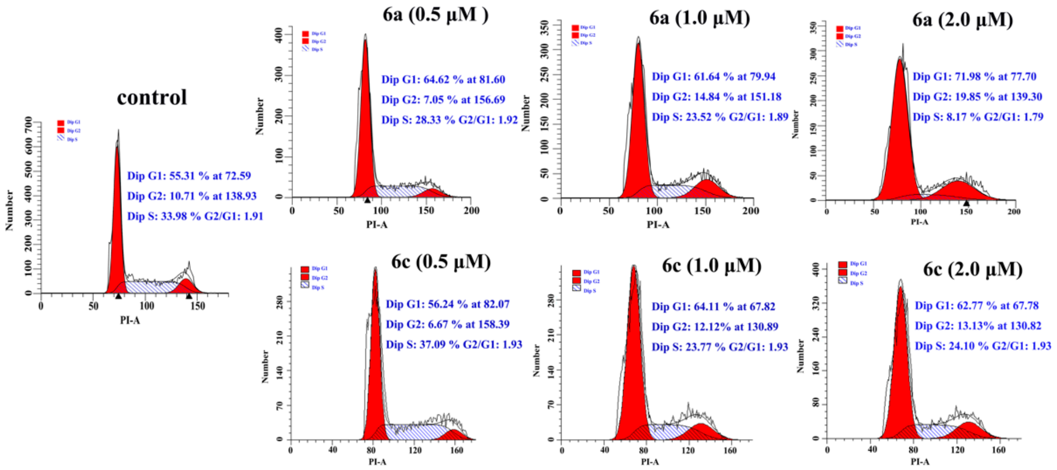

Figure S2: Cell cycle distribution of T-24 cells exposed to the **6a** and **6c** (0.5, 1, 2 µM) for 24 h. Effects on cell cycle progression of these compounds were examined according to the procedures described in the experimental section.

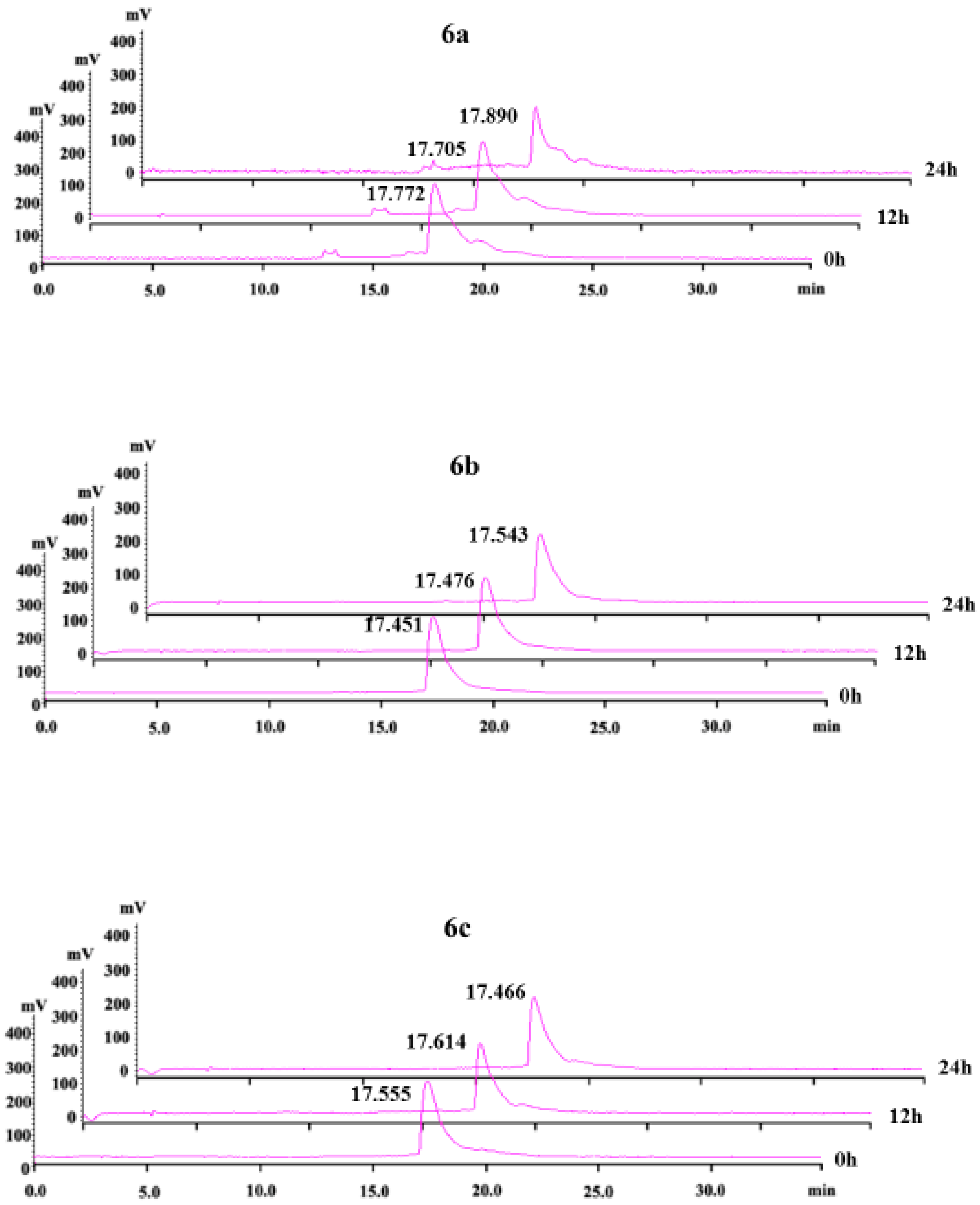

Figure S3: HPLC chromatograms for **6a**, **6b** and **6c** in aqueous solution (1 mg/mL) in the time courses of 0 h, 12 h and 24h, respectively. Column: reversed-phase C18 column (Agilent 5 TC-C18 250*4.6 mm.). Column temperature: 35°C. Mobile phase: CH_3OH/H_2O (30:70). Flow rate: 1.0 ml/min. Injection volume: 20 μM.

3. ^{1}H NMR, ^{13}CNMR and HRMS of compounds 6a-6c.

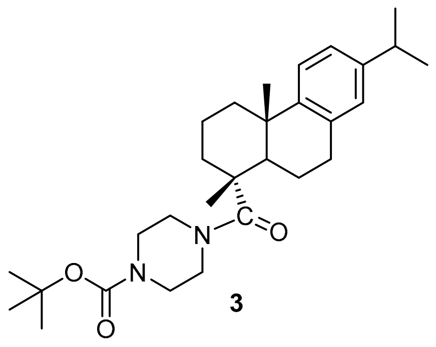

3

tert-butyl 4-((1R,4aS)-7-isopropyl-1,4a-dimethyl-1,2,3,4,4a,9,10,10a-octahydrophenanthrene-1-carbonyl)piperazine-1-carboxylate (3).Compound 1 (1 equivalent, 15 mmol) was dissolved in dichloromethane (100 mL), and excess oxalyl chloride (3.5 equivalent, 52.5 mmol) was added. After the reaction, the oxalyl chloride was removed, Boc-piperazine (1.2 equivalent, 18 mmol) was dissolved in the reaction solution, and the product 3 was obtained by separation and purification. It is a white solid with a yield of 82.3%. ^{1}H NMR (400 MHz, $CDCl_3$) δ 7.13 (d, *J* = 8.2 Hz, 1H), 6.96 (d, *J* = 8.1 Hz, 1H), 6.87 (s, 1H), 3.61 (dd, *J* = 22.9, 12.5 Hz, 4H), 3.40-3.30 (m, 4H), 2.92 (d, *J* = 18.4 Hz, 1H), 2.85 (s, 1H), 2.79 (s, 1H), 2.27 (t, *J* = 11.2 Hz, 2H), 1.75 (dd, *J* = 24.8, 18.7 Hz, 6H), 1.58 (s, 1H), 1.43 (s, 9H), 1.32 (s, 3H), 1.22 (s, 3H), 1.20 (s, 3H), 1.19 (s, 3H). ^{13}C NMR (101 MHz, $CDCl_3$) δ 177.59, 155.05, 147.24, 145.71, 135.15, 127.10, 124.20, 123.81, 80.63, 53.43, 46.97, 45.40, 37.66, 37.46, 35.50, 33.45, 30.61, 28.40, 25.50. HRMS (m/z) (ESI): $C_{29}H_{44}N_2O_3$ $[M+H]^+$ calcd for: 469.3352 found: 469.3457.

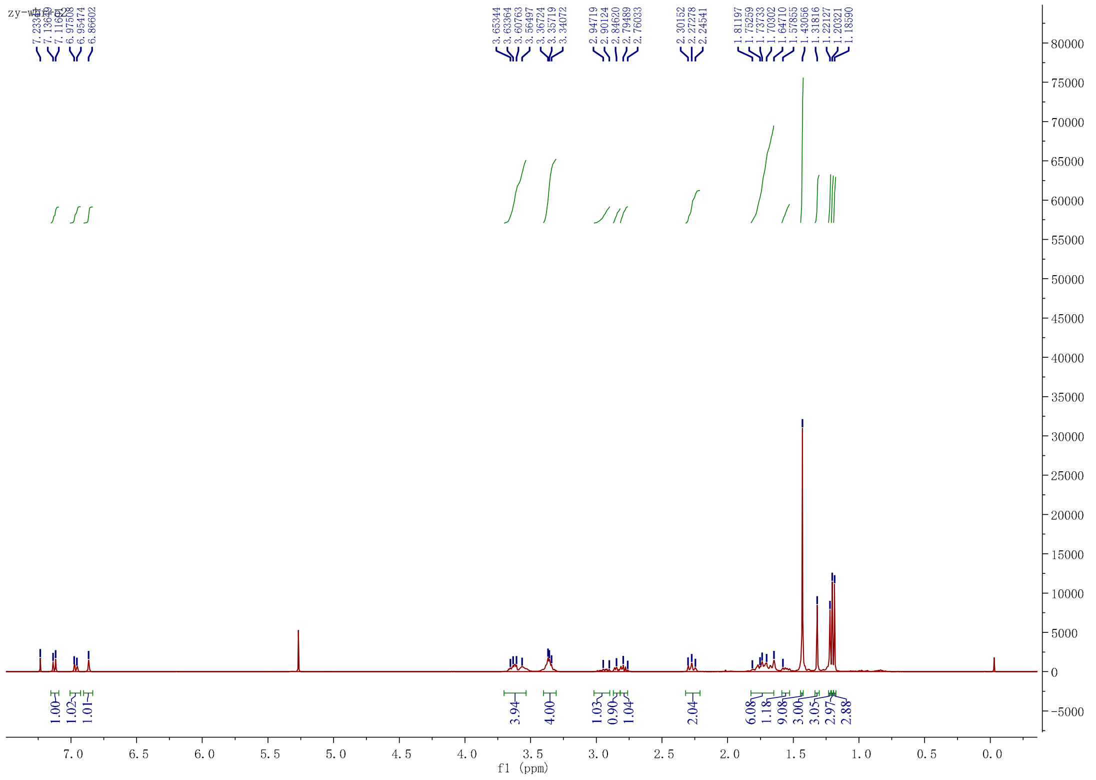
zy-wbr
7.23340
7.13639
7.11661
6.97308
6.95474
6.86602
3.65344
3.63364
3.60763
3.56497
3.36724
3.35719
3.34072
2.94719
2.90124
2.84620
2.79989
2.76033
2.30152
2.27278
2.24541
1.81197
1.75259
1.73733
1.70302
1.64710
1.57865
1.43056
1.31816
1.22127
1.20321
1.18390
1.00
1.02
1.01
3.94
4.00
1.03
0.90
1.04
2.04
6.08
1.18
9.08
3.00
3.05
2.97
2.88
f1 (ppm)
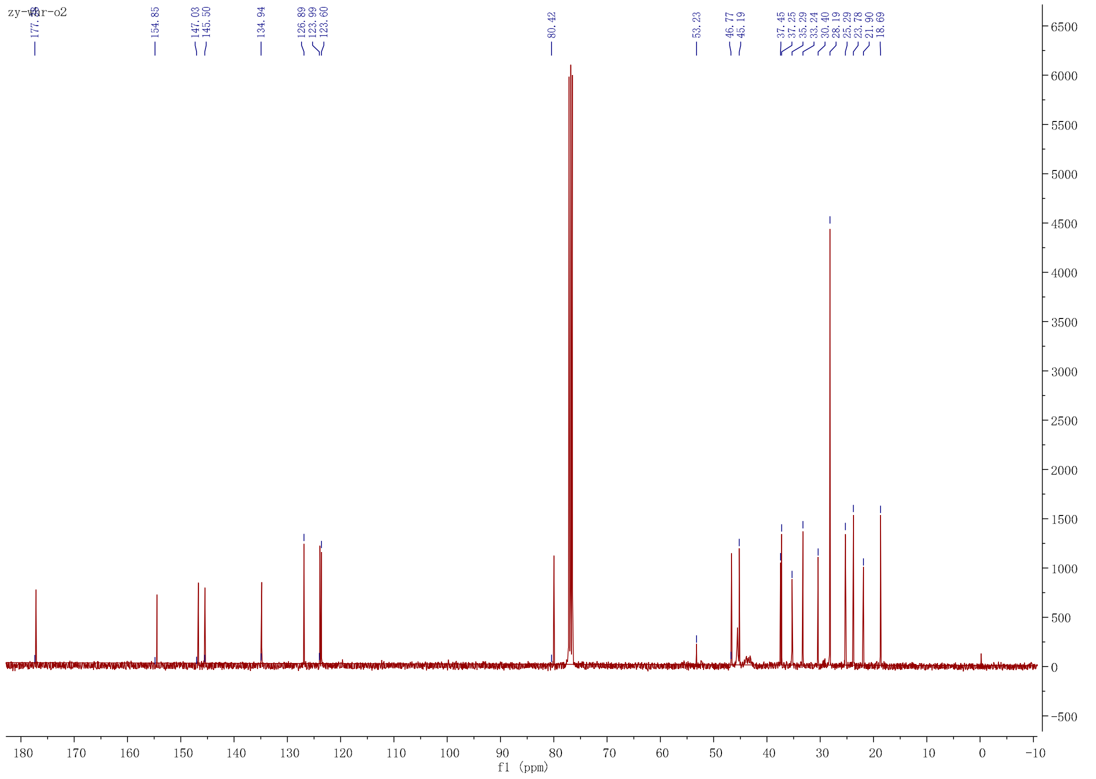
zy-wbr-c2
177.48
154.85
147.03
145.50
134.94
126.89
123.99
123.60
80.42
53.23
46.77
45.19
37.45
37.25
35.29
33.24
30.40
28.19
25.29
23.78
21.90
18.69
f1 (ppm)

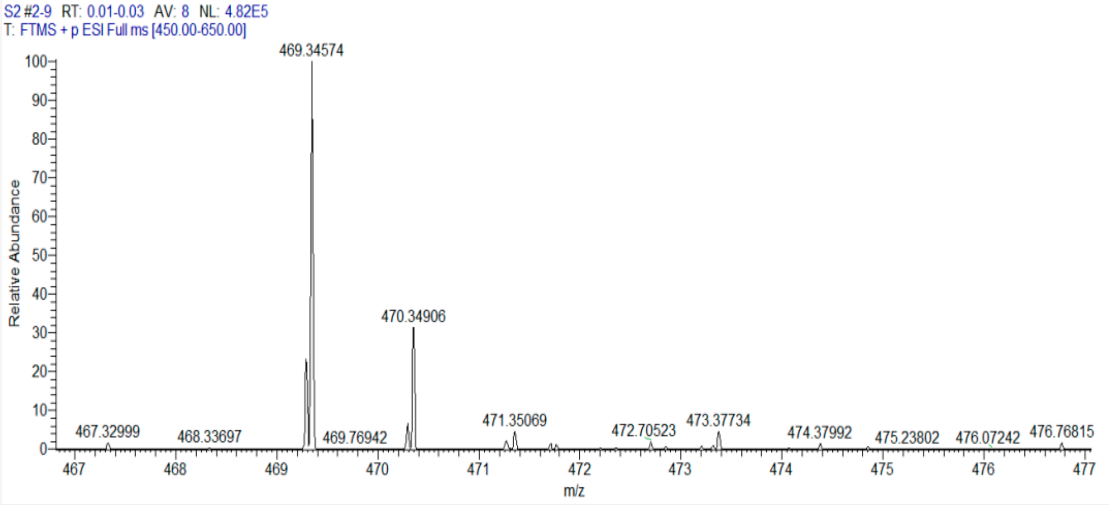
S2 #2-9 RT: 0.01-0.03 AV: 8 NL: 4.82E5
T: FTMS + p ESI Full ms [450.00-650.00]
469.34574
470.34906
467.32999
468.33697
469.76942
471.35069
472.70523
473.37734
474.37992
475.23802
476.07242
476.76815
Relative Abundance
m/z

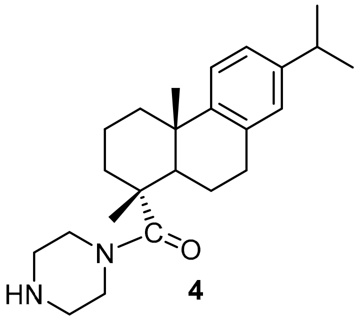

4

((1R,4aS)-7-isopropyl-1,4a-dimethyl-1,2,3,4,4a,9,10,10a-octahydrophenanthren-1-yl)(piperazin-1-yl)methanone (4). Compound 3 (1 equivalent, 15 mmol) was dissolved in dichloromethane (100 mL), and excess trifluoroacetic acid (3.5 equivalents, 52.5 mmol) was added. After the reaction, extract and adjust the pH to neutral to obtain product 4. It is a white solid with a yield of 76.5%. ^{1}H NMR (400 MHz, $CDCl_3$) δ 7.14 (d, *J* = 8.2 Hz, 1H), 6.99 (d, *J* = 9.6 Hz, 1H), 6.89 (s, 1H), 3.98 (dd, *J* = 32.2, 14.9 Hz, 4H), 3.17 (s, 4H), 2.91 (dd, *J* = 50.3, 23.3 Hz, 3H), 2.27 (t, *J* = 11.6 Hz, 2H), 1.76 (dd, *J* = 22.3, 16.6 Hz, 4H), 1.67 - 1.39 (m, 3H), 1.33 (s, 3H), 1.24 (s, 3H), 1.22 (s, 3H), 1.21 (s, 3H). ^{13}C NMR (101 MHz, $CDCl_3$) δ 177.49 (s), 146.60 (s), 145.84 (s), 134.85 (s), 127.13 (s), 124.01 (d, *J* = 18.7 Hz), 46.80 (s), 45.49 (s), 43.55 (s), 42.62 (s), 37.49 (d, *J* = 19.5 Hz), 35.75 (s), 33.46 (s), 30.45 (s), 25.39 (s), 23.98 (d, *J* = 2.9 Hz), 22.16 (s), 18.66 (d, *J* = 13.8 Hz). HRMS (m/z) (ESI): $C_{24}H_{36}N_2O$ $[M+H]^+$ calcd for: 369.2828 found: 369.2919.

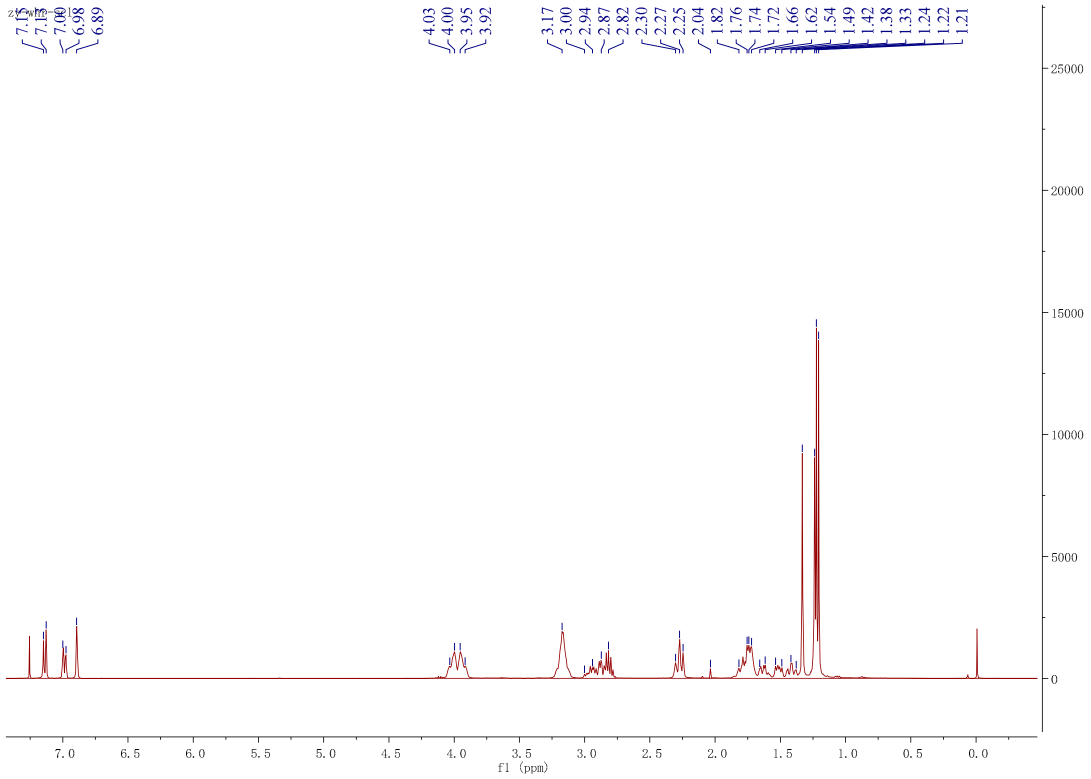

.

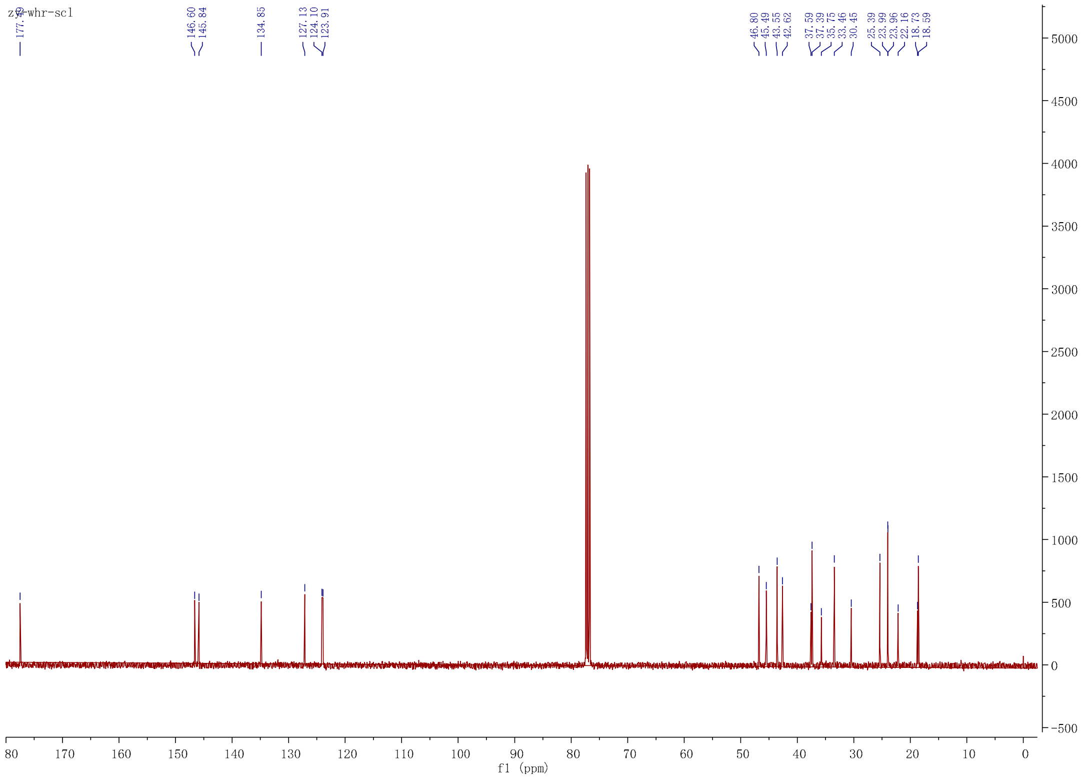

zjq-whr-scl
177.49
146.60
145.84
134.85
127.13
124.10
123.91
46.80
45.49
43.55
42.62
37.59
37.39
35.75
33.46
30.45
25.39
23.99
23.96
22.16
18.73
18.59
f1 (ppm)

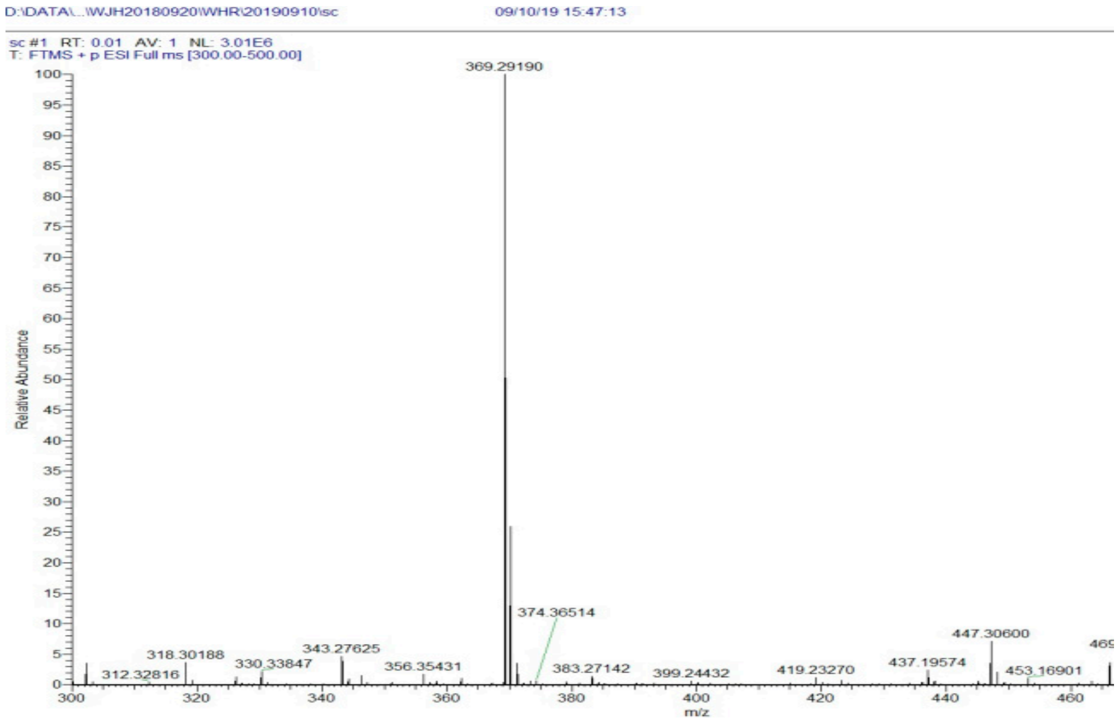

D:\DATA\...\WJH20180920\WHR\20190910\sc
09/10/19 15:47:13
sc #1 RT: 0.01 AV: 1 NL: 3.01E6
T: FTMS + p ESI Full ms [300.00-500.00]
369.29190
374.36514
318.30188
343.27625
330.33847
356.35431
312.32816
383.27142
399.24432
419.23270
437.19574
447.30600
453.16901
Relative Abundance
m/z

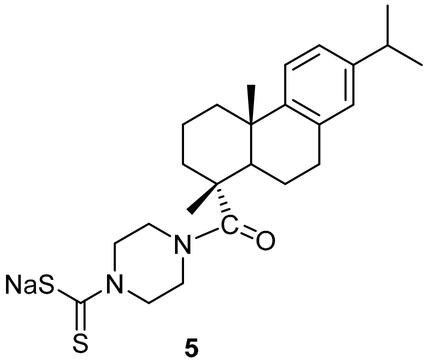

5

sodium 4-((1R,4aS)-7-isopropyl-1,4a-dimethyl-1,2,3,4,4a,9,10,10a-octahydrophenanthrene-1-carbonyl)piperazine-1-carbodithioate (5). Compound 4 (1 equivalent, 15 mmol) was dissolved in dichloromethane (100 mL), and sodium hydroxide solution (1 equivalent, 15 mmol) and excess CS2 were added. Separate and purify after the reaction to obtain purified product 5. It is a white solid with a yield of 63.2%. ^{1}H NMR (400 MHz, $CDCl_3$) δ 7.15 (d, *J* = 8.2 Hz, 1H), 6.99 (dd, *J* = 8.2, 1.6 Hz, 1H), 6.89 (s, 1H), 4.14 - 4.06 (m, 1H), 3.79 (dd, *J* = 18.4, 13.4 Hz, 5H), 3.04 -2.73 (m, 3H), 2.30 (d, *J* = 11.8 Hz, 2H), 2.04 (s, 1H), 1.84-1.66 (m, 5H), 1.56 (dd, *J* = 12.7, 6.9 Hz, 1H), 1.44 (dd, *J* = 13.3, 10.4 Hz, 1H), 1.35 (s, 3H), 1.29 - 1.24 (m, 4H), 1.23 (d, *J* = 3.3 Hz, 3H), 1.21 (s, 3H). ^{13}C NMR (101 MHz, $CDCl_3$) δ 177.49, 146.60, 145.84, 134.85, 127.13, 124.10, 123.91, 46.80, 45.49, 43.55, 42.62, 37.59, 37.39, 35.75, 33.46, 30.45, 25.39, 23.99, 23.96, 22.16, 18.73, 18.59. HRMS (m/z) (ESI): $C_{25}H_{35}N_2NaOS_2$ $[M+H]^+$ calcd for: 489.2088, found: 489.21.

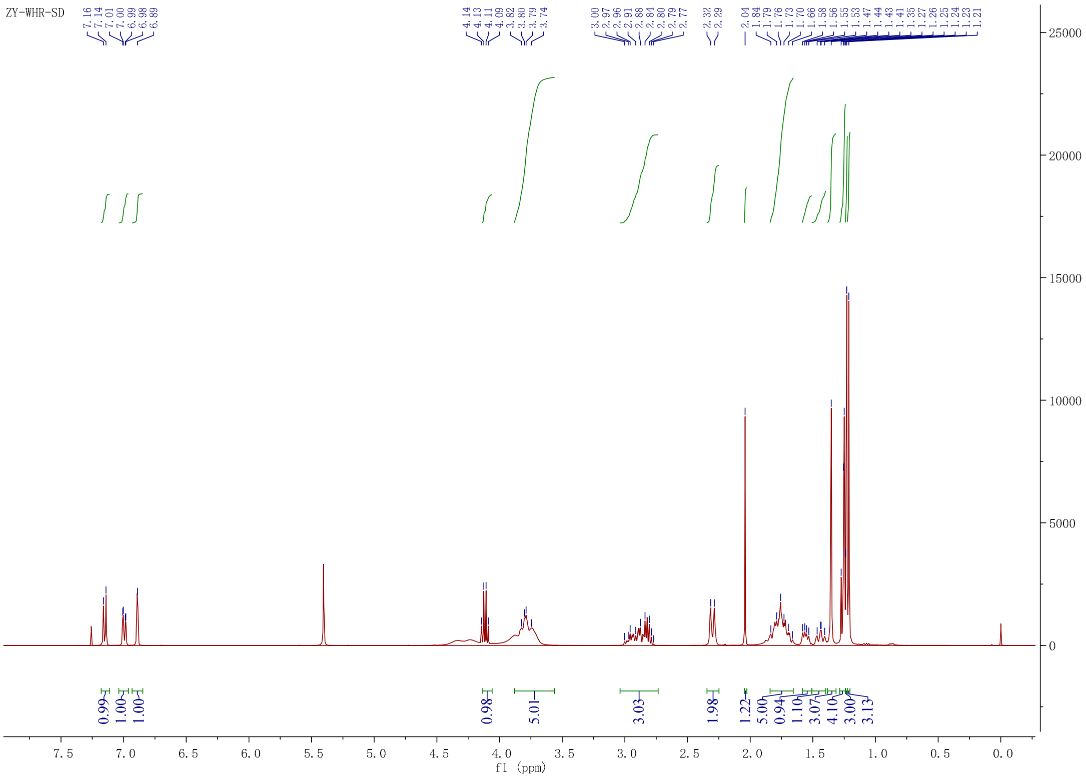

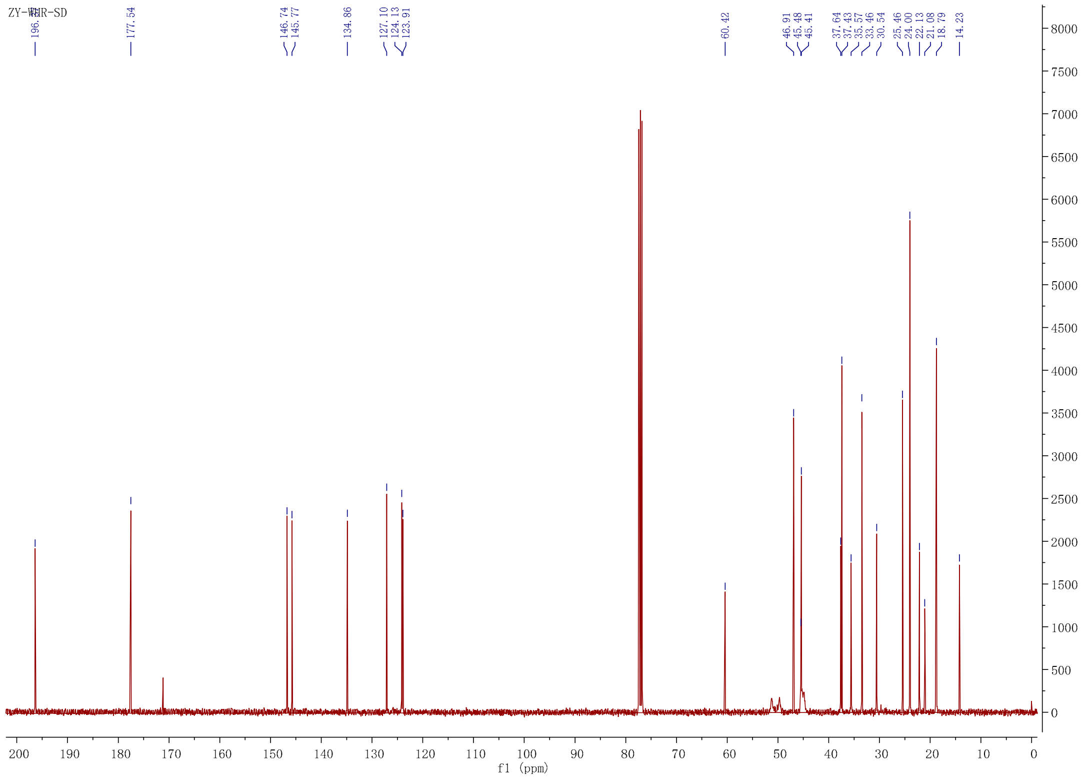
ZY-WHR-SD
196.
177.54
146.74
145.77
134.86
127.10
124.13
123.91
60.42
46.91
45.48
45.41
37.64
37.43
35.57
33.46
30.54
25.46
24.00
22.13
21.08
18.79
14.23
f1 (ppm)

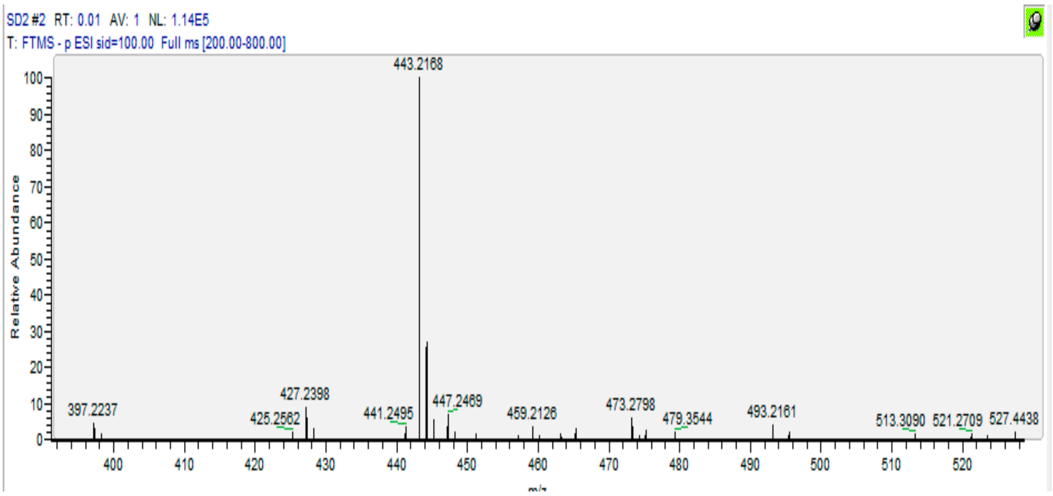
SD2 #2 RT: 0.01 AV: 1 NL: 1.14E5
T: FTMS - p ESI sid=100.00 Full ms [200.00-800.00]
443.2168
427.2398
397.2237
425.2562
441.2495
447.2469
459.2126
473.2798
479.3544
493.2161
513.3090
521.2709
527.4438
Relative Abundance

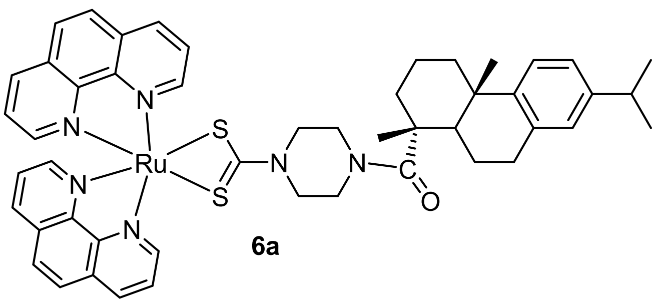

6a

(6a). Yield 56.2%. ^{1}H NMR (400 MHz, DMSO) δ 9.87 (d, *J* = 5.3 Hz, 2H), 8.83 (d, *J* = 8.1 Hz, 2H), 8.50 (d, *J* = 8.2 Hz, 2H), 8.37 (d, *J* = 8.9 Hz, 2H), 8.26 (dd, *J* = 15.3, 7.9 Hz, 4H), 7.93 (d, *J* = 4.1 Hz, 2H), 7.54 – 7.48 (m, 2H), 7.13 (dd, *J* = 7.0, 3.8 Hz, 1H), 6.97 – 6.67 (m, 2H), 3.86 (s, 4H), 3.65 (s, 4H), 2.76 (s, 3H), 2.12 (ddd, *J* = 70.1, 45.3, 15.8 Hz, 3H), 1.74 (dd, *J* = 26.6, 18.6 Hz, 3H), 1.67 – 1.36 (m, 6H), 1.26 (d, *J* = 8.1 Hz, 3H), 1.13 (dd, *J* = 10.5, 4.6 Hz, 6H). ^{13}C NMR (101 MHz, DMSO) δ 212.75, 176.97, 155.33 , 152.61 , 148.93 , 148.55 , 145.43 , 135.33, 134.86, 130.62, 130.29, 128.36, 127.90, 126.97 , 126.49, 125.59, 124.64, 124.10, 46.81, 46.31, 45.58, 44.76, 37.39, 33.34, 30.48, 29.50, 25.64, 24.38, 18.89, 18.43, 14.38. Anal. Calcd for [$C_{49}H_{51}N_6ORuS_2$]Cl (940.2298): C, 62.57; H, 5.46; N, 8.93. Found: C, 62.50; H, 5.39; N, 9.02. HRMS (m/z) (ESI) 905.2606 [$C_{49}H_{51}N_6ORuS_2$]$^+$.

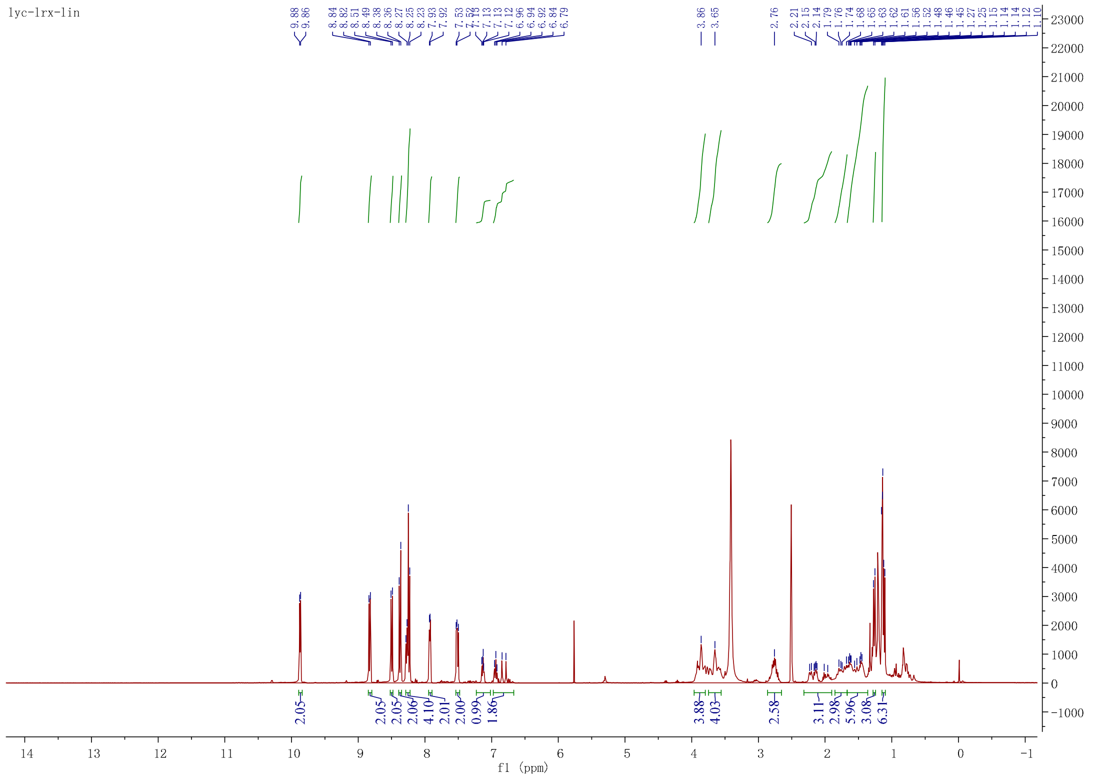

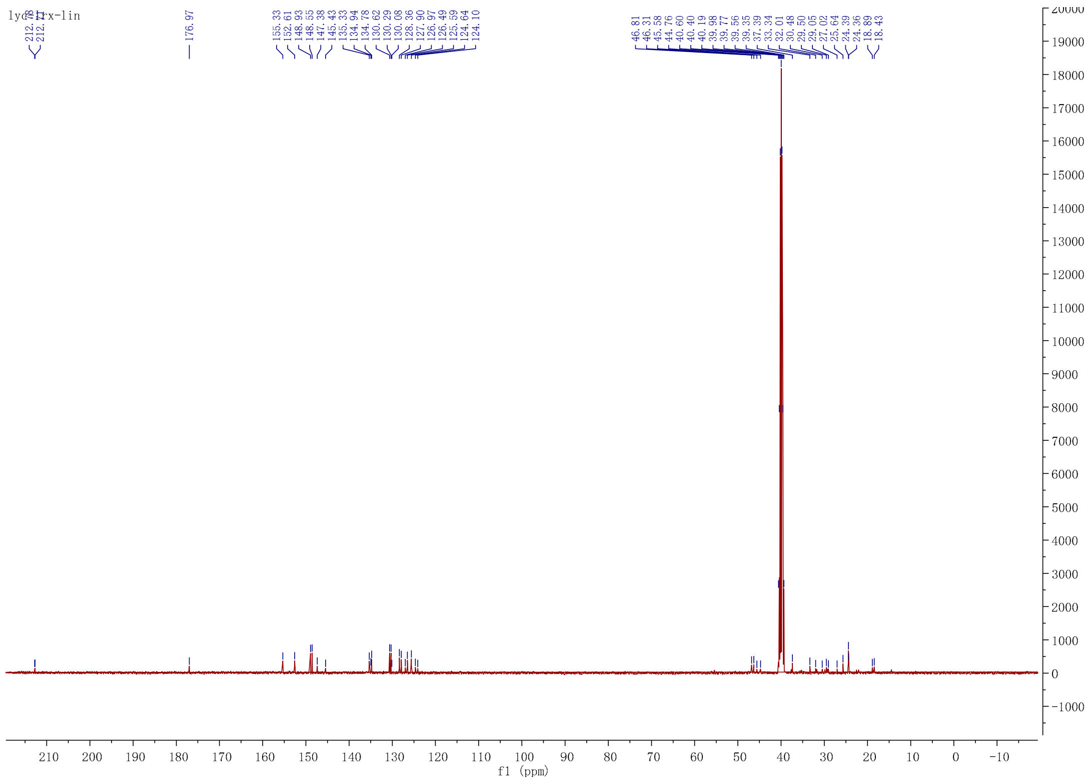
lyd-trx-lin
212.78
212.77
176.97
155.33
152.61
148.93
148.55
147.38
145.43
135.33
134.94
134.78
130.62
130.29
130.08
128.36
127.90
126.97
126.49
125.39
124.64
124.10
46.81
46.31
45.38
44.76
40.60
40.40
40.19
39.98
39.77
39.56
39.35
37.39
33.34
32.01
30.48
29.50
29.05
27.02
25.64
24.39
24.36
18.89
18.43
f1 (ppm)

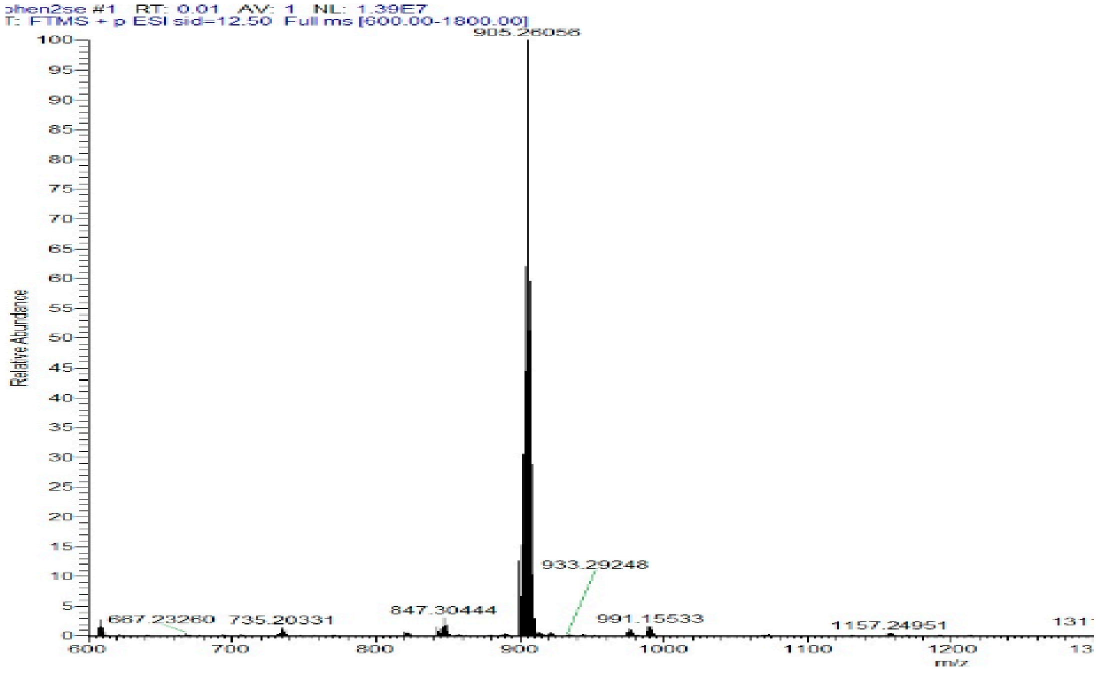
phen2se #1 RT: 0.01 AV: 1 NL: 1.39E7
T: FTMS + p ESI sid=12.50 Full ms [600.00-1800.00]
905.26056
933.29248
687.23260
735.20331
847.30444
991.15533
1157.24951
Relative Abundance
m/z

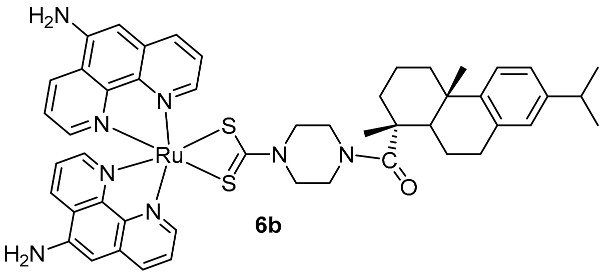

6b

(6b). Yield 53.2%. ^{1}H NMR (400 MHz, DMSO) δ 10.57 (s, 2H), 9.82 (d, *J* = 5.2 Hz, 1H), 9.44 – 9.35 (m, 1H), 9.00 (s, 1H), 8.68 (d, *J* = 8.4 Hz, 1H), 8.32 (dd, *J* = 8.0, 2.8 Hz, 1H), 8.03 – 7.90 (m, 2H), 7.82 (s, 1H), 7.47 – 7.35 (m, 2H), 7.29 – 7.21 (m, 1H), 7.18 – 7.09 (m, 2H), 7.02 – 6.83 (m, 4H), 6.76 (s, 2H), 3.37 (s, 8H), 2.86 – 2.67 (m, 3H), 1.29 (d, *J* = 8.7 Hz, 3H), 1.26 (s, 3H), 1.23 (s, 6H), 1.21 (s, 6H), 1.19 (s, 3H). ^{13}C NMR (101 MHz, $CDCl_3$) δ 204.22, 168.66, 155.06, 153.79, 149.47, 135.07, 130.43, 127.78, 127.36, 106.54, 106.08, 100.00, 56.12, 50.44, 41.49, 41.02, 37.40, 33.31, 33.03, 28.27, 27.05, 22.34, 11.76, 10.17, 4.03. Anal. Calcd for $[C_{49}H_{53}N_8ORuS_2]Cl$ (970.2516): C, 60.63; H, 5.50; N, 11.54. Found: C, 62.55; H, 5.58; N, 11.46. HRMS (m/z) (ESI) 935.2820 $[C_{49}H_{53}N_8ORuS_2]^+$.

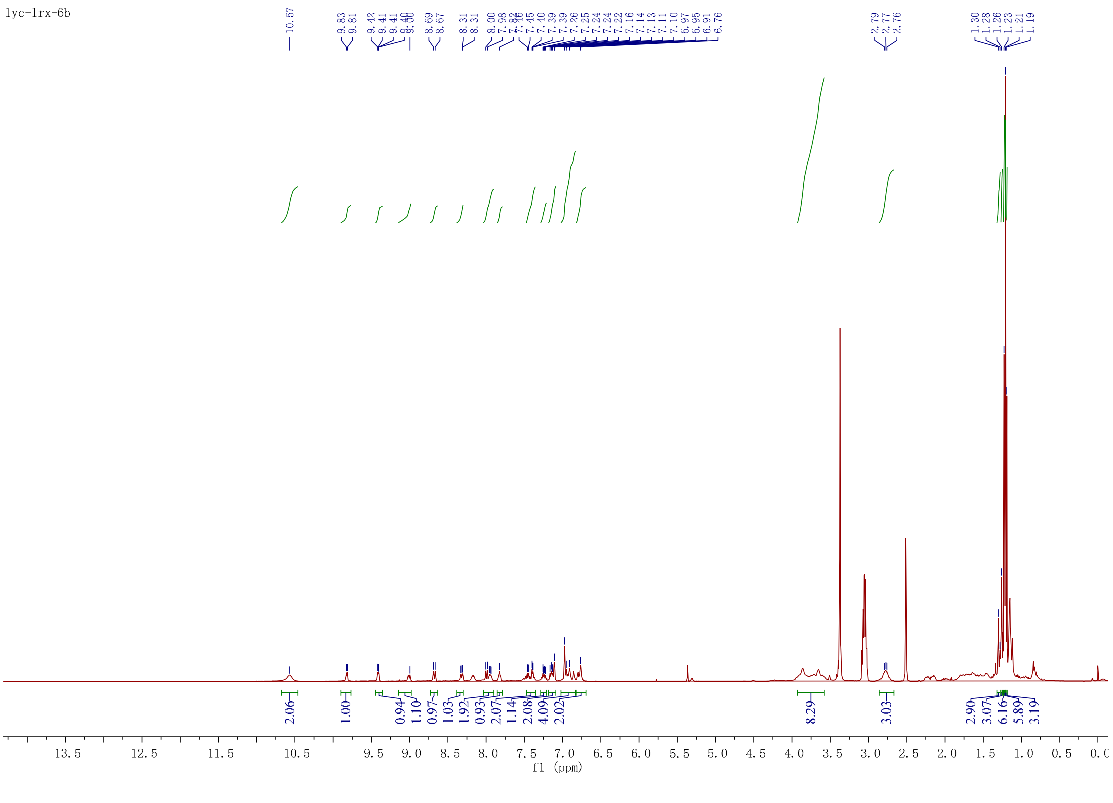

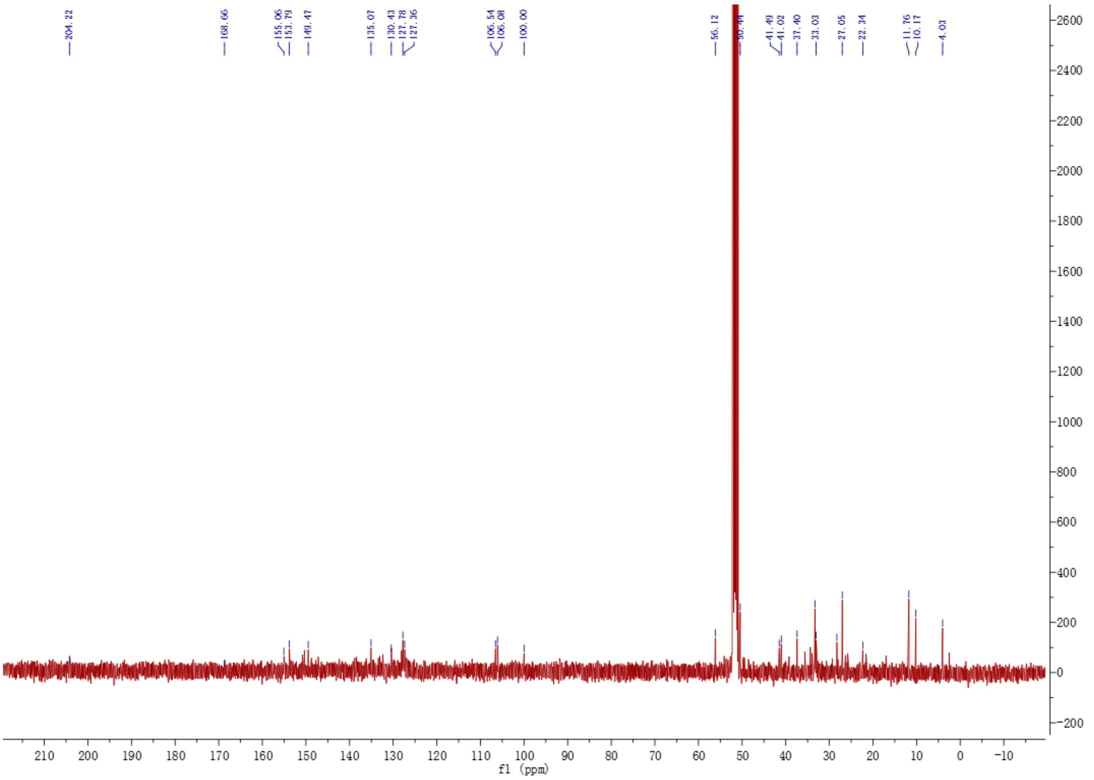

204.22
168.66
155.06
153.79
149.47
135.07
130.43
127.78
127.36
106.54
106.08
100.00
56.12
50.44
41.49
41.02
37.40
33.03
27.05
22.34
11.76
10.17
4.03
f1 (ppm)

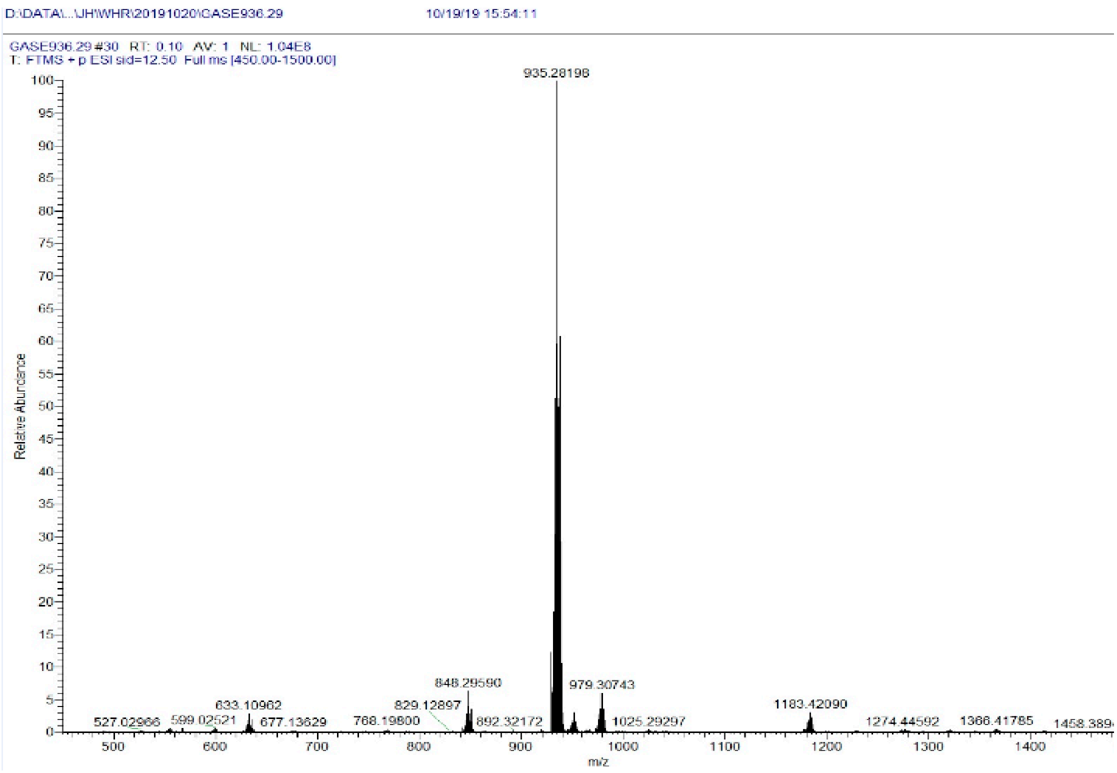

D:\DATA\...\JH\WHR\20191020\GASE936.29
10/19/19 15:54:11
GASE936.29 #30 RT: 0.10 AV: 1 NL: 1.04E8
T: FTMS + p ESI sid=12.50 Full ms [450.00-1500.00]
935.28198
848.29590
979.30743
633.10962
829.12897
1183.42090
527.02966
599.02521
677.13629
768.19800
892.32172
1025.29297
1274.44592
1366.41785
Relative Abundance
m/z

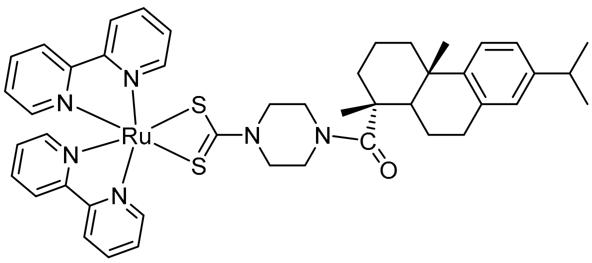

(6c). Yield 55.3%. ^{1}H NMR (400 MHz, $CDCl_3$) δ 9.53 (d, *J* = 5.6 Hz, 2H), 8.68 (d, *J* = 6.0 Hz, 2H), 8.57 (d, *J* = 8.1 Hz, 2H), 8.08 (s, 2H), 7.80 (s, 2H), 7.66 (ddd, *J* = 8.9, 4.6, 2.5 Hz, 2H), 7.56 (d, *J* = 5.3 Hz, 2H), 7.12 (dd, *J* = 7.4, 5.7 Hz, 3H), 6.97 (s, 1H), 6.87 (d, *J* = 8.5 Hz, 1H), 4.02 – 3.66 (m, 8H), 2.96 – 2.77 (m, 3H), 1.86 – 1.60 (m, 6H), 1.34 (d, *J* = 5.3 Hz, 3H), 1.23 (d, *J* = 3.2 Hz, 6H), 1.19 (d, *J* = 6.6 Hz, 6H). ^{13}C NMR (101 MHz, $CDCl_3$) δ 214.28, 177.73, 177.69, 158.15, 158.00, 153.71, 153.70, 150.90, 146.76, 146.74, 145.76, 145.74, 136.01, 135.44, 134.91, 127.06, 126.67, 126.00, 24.17, 123.85, 123.84, 123.73, 46.85, 46.83, 46.42, 45.51, 44.93, 37.62, 37.43, 35.76, 33.43, 30.57, 29.70, 5.47, 23.98, 22.19, 18.80, 18.73, 14.14. Anal. Calcd for $[C_{45}H_{51}N_6ORuS_2]Cl$ (892.2298): C, 60.55; H, 5.76; N, 9.42. Found: C, 60.62; H, 5.68; N, 9.36. HRMS (m/z) (ESI) 857.2607 $[C_{45}H_{51}N_6ORuS_2]^+$.

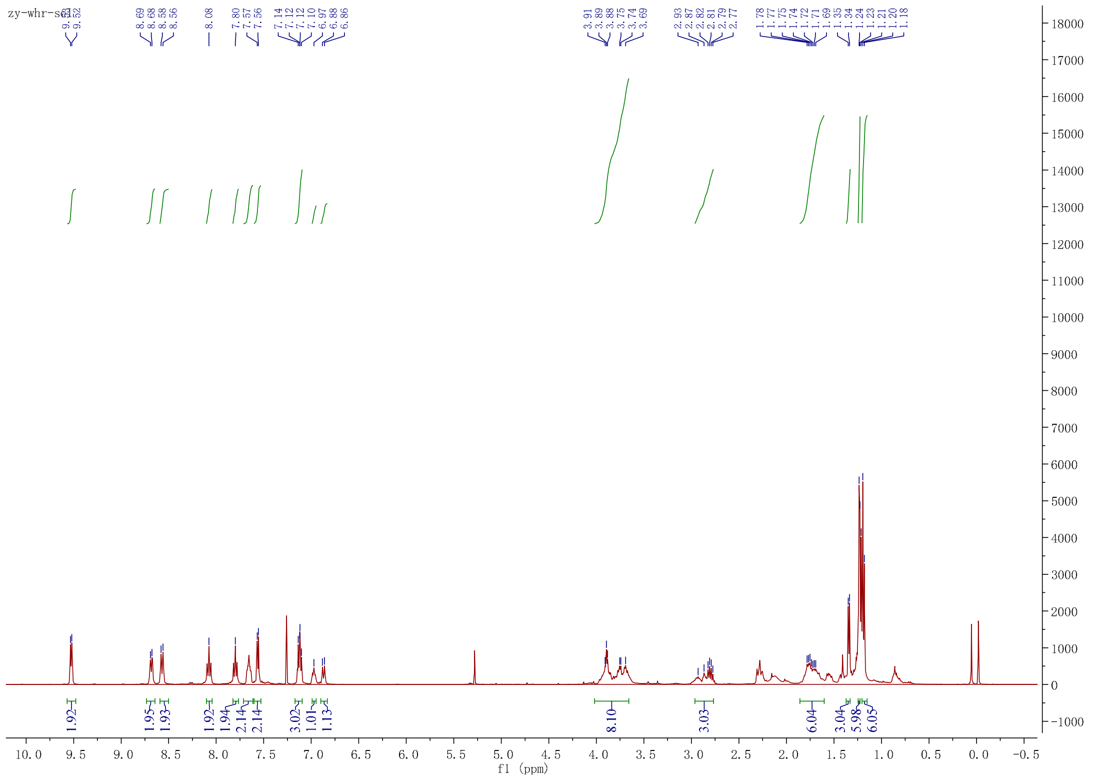

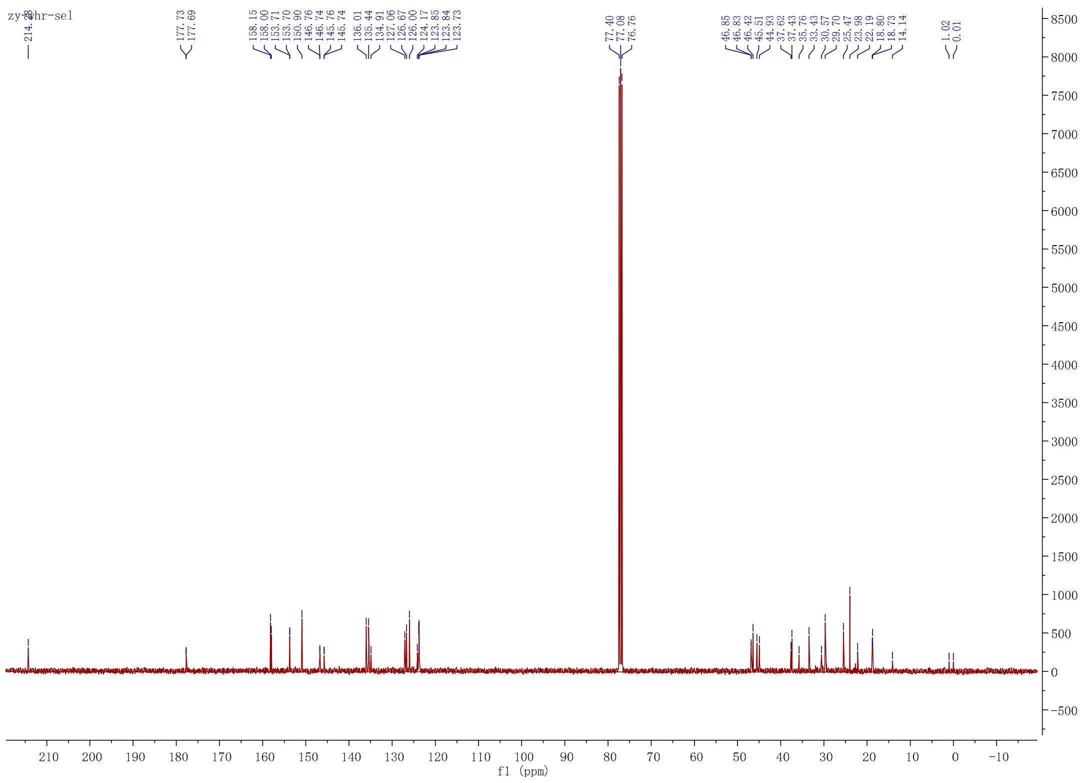
zy-whr-sel
214.28
177.73
177.69
158.15
158.00
153.71
153.70
150.90
146.76
146.74
145.76
145.74
136.01
135.44
134.91
127.06
126.67
126.00
124.17
123.85
123.84
123.73
77.40
77.08
76.76
46.85
46.83
46.42
45.51
44.93
37.62
37.43
35.76
33.43
30.57
29.70
25.47
23.98
22.19
18.80
18.73
14.14
1.02
0.01
f1 (ppm)

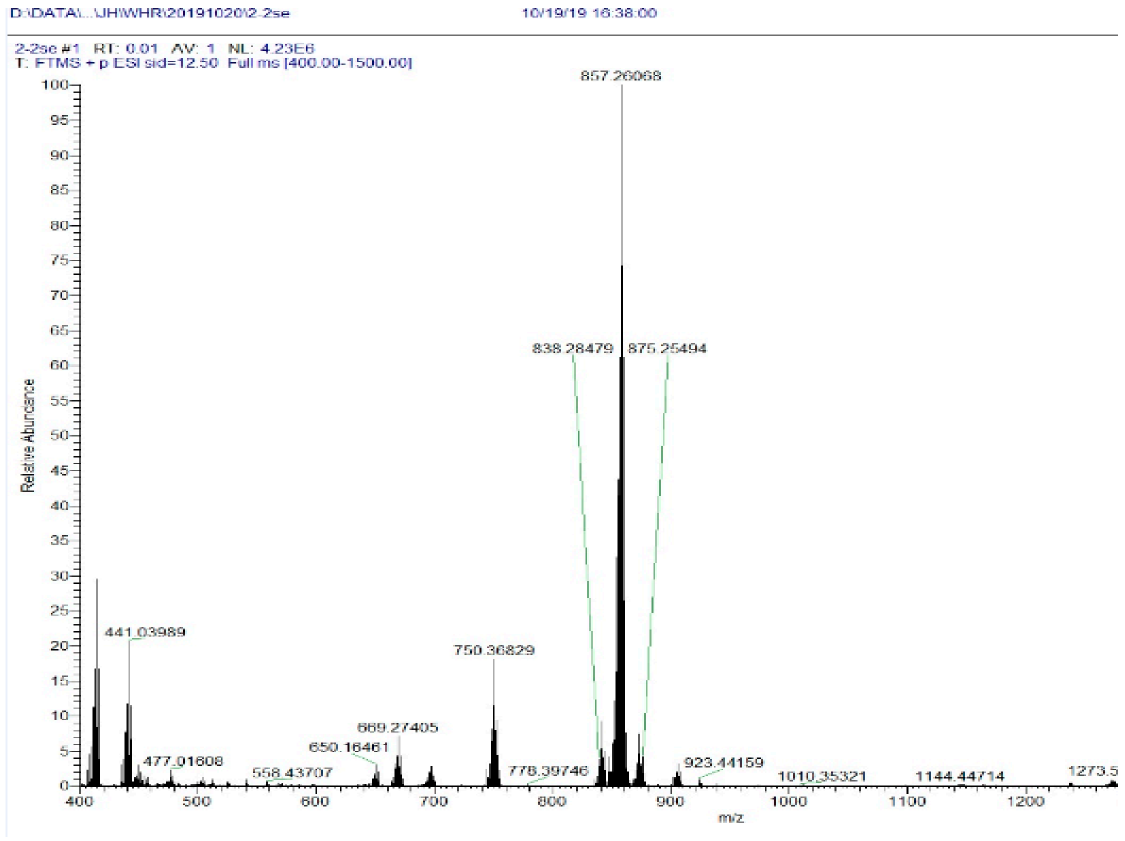
D:\DATA\...\JH\WHR\20191020\2-2se
10/19/19 16:38:00
2-2se #1 RT: 0.01 AV: 1 NL: 4.23E6
T: FTMS + p ESI sid=12.50 Full ms [400.00-1500.00]
857.26068
838.28479
875.25494
441.03989
750.36829
669.27405
650.16461
477.01608
558.43707
778.39746
923.44159
1010.35321
1144.44714
1273.5
Relative Abundance
m/z
